# Reducing the *V*_oc_ Loss of Hole Transport Layer-Free Carbon-Based Perovskite Solar Cells via Dual Interfacial Passivation

**DOI:** 10.1007/s40820-025-01775-4

**Published:** 2025-05-19

**Authors:** Xian Zhang, Fangzhou Liu, Yan Guan, Yu Zou, Cuncun Wu, Dongchang Shi, Hongkai Zhang, Wenjin Yu, Dechun Zou, Yangyang Zhang, Lixin Xiao, Shijian Zheng

**Affiliations:** 1 Key Laboratory of Materials Laminating Fabrication and Interface Control Technology of Tianjin, School of Materials Science and Engineering, Hebei University of Technology, Tianjin, 300401 People’s Republic of China; 2 College of Chemistry and Molecular Engineering, Peking University, Beijing, 100871 People’s Republic of China; 3 State Key Laboratory for Mesoscopic Physics and Department of Physics, Peking University, Beijing, 100871 People’s Republic of China

**Keywords:** Perovskite solar cells, Carbon electrode, Hole transport layer-free, Open-circuit voltage, Indoor photovoltaic

## Abstract

The hole transport layer (HTL)-free carbon-based perovskite solar cells (C-PSCs) are promising for commercialization owing to their excellent operational stability and simple fabrication process. However, the power conversion efficiencies (PCE) of C-PSCs are inferior to the metal electrode-based devices due to their open-circuit voltage (*V*_oc_) loss. Herein, time-resolved confocal photoluminescence microscopy reveals that grain boundary defects at the perovskite/carbon interface are very likely to function as nonradiative recombination centers in HTL-free C-PSCs. A versatile additive Li_2_CO_3_ is used to modify the conformal tin oxide electron transport layer for HTL-free C-PSCs. Li_2_CO_3_ modification can result in enhanced charge extraction and optimized energy alignment at electron transport layer/perovskite interface, as well as suppressed defects at perovskite top surface due to Li_2_CO_3_-induced formation of PbI_2_ crystallites. Such dual interfacial passivation ultimately leads to significantly improved *V*_oc_ up to 1.142 V, which is comparable to the metal electrode-based devices with HTL. Moreover, a record-high PCE of 33.2% is achieved for Li_2_CO_3_-modified C-PSCs under weak light illumination conditions, demonstrating excellent indoor photovoltaic performance. This work provides a practical approach to fabricate low-cost, highly efficient carbon-based perovskite solar cells.

**Supplementary Information:**

The online version contains supplementary material available at 10.1007/s40820-025-01775-4.

## Highlights

- Li_2_CO_3_ is used to modify conformal SnO_2_ as electron transport layer for hole transport layer-free carbon-based perovskite solar cells (C-PSCs).
- CO_3_^2−^ can induce MA release from the perovskite layer, resulting in PbI_2_ at the grain boundary of top interface of perovskite film which can passivate the grain boundary and the top surface defects.
- The Li_2_CO_3_-modified C-PSC exhibits a high power conversion efficiency (PCE) of 19.1%, with a *V*_oc_ of 1.142 V. A record-high PCE of 33.2% is obtained under weak light-emitting diode illumination (2000 lx, 3000 K).

**Supplementary Information:**

The online version contains supplementary material available at 10.1007/s40820-025-01775-4.

## Graphical abstract

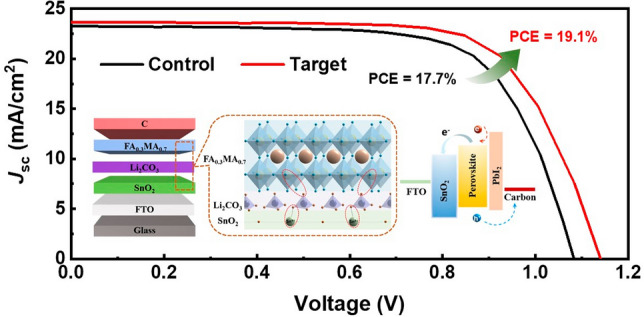

## Introduction

Organic–inorganic hybrid perovskite solar cells (PSCs) have been outstanding among the next-generation photovoltaic technologies in the past decade due to their ease of fabrication and excellent photovoltaic properties, such as high optical absorption coefficients, long carrier diffusion length, and low exciton binding energy [1–5]. The certified power conversion efficiencies (PCE) of PSCs have reached up to 27.0% [6], which is close to the efficiencies achieved by the best-performing monocrystalline silicon solar cells. While the soaring efficiencies make these technologies highly attractive for future commercialization, the long-term stability of PSCs remains a major concern for their practical applications [7–10].

Hole transport layer (HTL)-free carbon-based perovskite solar cells (C-PSCs) are considered as a promising candidate for commercialization due to their extraordinary operational stability and cost-effective fabrication processes [11–15]. However, the PCE of HTL-free C-PSCs lags significantly behind those of the PSCs with HTL, accompanied with severe open-circuit voltage (*V*_oc_) loss [15–17]. Several strategies have been employed to improve the efficiency of HTL-free C-PSCs, for instance, enhancing electron/hole extraction [13], optimizing energy alignment [18, 19], passivating interfacial trap states [10, 20–22], and suppressing ion migration [23]. One of the primary reasons for the large *V*_oc_ loss in the HTL-free C-PSCs is the nonradiative recombination at both top and buried bottom interfaces. Many previous studies on HTL-free C-PSCs have been focusing on the interface of perovskite absorbers and carbon electrodes [14, 18, 24, 25]. For instance, enhanced interfacial energy-level alignment in the HTL-free C-PSCs can be achieved by depositing a thin layer of poly(ethylene oxide) at the perovskite/carbon interface, and the optimized devices showed an increased PCE from 12.2% to 14.9% [18]. Employing a 2D perovskite passivating layer as an electron blocking layer (EBL) atop the 3D perovskite absorber has also been demonstrated to substantially suppress the interfacial recombination loss [26, 27]. For example, an octylammonium-based 2D perovskite EBL can effectively passivate the surface trap states and block the undesirable electron transfer, which enables HTL-free C-PSCs with a PCE of 18.5% and a *V*_oc_ of 1.05 V [14]. Furthermore, an optimal balance between defect passivation, energy-level structure, and charge transport is achieved with 2D perovskite passivating layer using tetradecylammonium cation, which leads to a record-high PCE of 20.4% (certified 20.1%) for HTL-free C-PSCs to date [28]. Other strategies address the improved material design of carbon electrodes to ameliorate the perovskite/carbon interface. A bilayer carbon electrode comprising a coherent layer and a conductive layer has been reported to facilitate efficient charge transfer at the perovskite/carbon interface [29]. A thin layer of oxidized multi-walled carbon nanotubes (O-MWCNTs) deposited on perovskite top surface during antisolvent dripping has been demonstrated to facilitate charge extraction and transport of HTL-free C-PSCs, while improving the hydrophobicity of the interface [30]. On the other hand, considerably less research efforts have been reported for optimization of electron transport layer (ETL) to enhance the charge transport at perovskite bottom interface, including the use of blade-coated fullerene ETL on amphiphilic silane-modified transparent conductive oxide substrates [31], as well as the [6, 6]-phenyl-C_61_-butyric acid methyl ester (PCBM)-modified nanoneedle-like TiO_2_ ETL [13]. Nevertheless, the significant *V*_oc_ loss remains challenging for further progress of HTL-free C-PSCs, which requires more research insights on the passivation strategies for both ETL/perovskite and perovskite/carbon interfaces.

In this work, taking into consideration that *V*_oc_ loss in HTL-free C-PSCs is profoundly linked to the defects at both ETL/perovskite interface and perovskite/carbon interface, we report Li_2_CO_3_ as a versatile surface modifier for conformal tin oxide (C-SnO_2_) ETL, and demonstrate the dual interfacial passivation via Li_2_CO_3_ modification. For the ETL/perovskite interface, enhanced charge extraction and transport is collectively achieved by reduced interfacial defects, optimized energy alignment, and increased ETL conductivity. Simultaneously, Li_2_CO_3_-induced formation of PbI_2_ crystallites at the grain boundaries can effectively passivate the defects at perovskite top surface. Such dual interfacial passivation via Li_2_CO_3_ modification leads to significantly reduced *V*_oc_ loss due to suppressed defects and improved charge extraction at both ETL/perovskite and perovskite/carbon interfaces. The Li_2_CO_3_-modified C-PSC exhibited an optimized *V*_oc_ up to 1.142 V with a PCE of 19.1%. More importantly, a record-high PCE of 33.2% was obtained under weak light LED illumination, indicating its great potential for low-energy harvesting applications.

## Experimental Section

### Materials

Fluorine-doped tin oxide (FTO)/glass substrates, lead iodide (PbI_2_, 99.99%), formamidinium iodide (HC(NH_2_)_2_I, FAI, 99.9%), methylammonium iodide (CH_3_NH_3_I, MAI, 99.9%), 4-tert-butylpyridine (TBP, 99%), lithium bis (trifluoromethylsulfonyl) imide (Li-TFSI, 99%) and 2,2’,7,7’-tetrakis(N,N-dip-methoxyphenyl-amine)-9,9’-spirobifluorene (Spiro-OMeTAD, 99.5%) were purchased from Advanced Election Technology Co., Ltd. Methylammonium chloride (MACl, 99.5%), cesium iodide (CsI, > 99.9%) and Poly[bis-(4-phenyl)(2,4,6-trimethylphenyl)amine] (PTAA, average Mn 6000–15,000) were purchased from Xi’an Polymer Light Technology Corp, China. Lithium carbonate (Li_2_CO_3_, 99.99%) was purchased from Shanghai Macklin Biochemical Co., Ltd. Urea (99.999%) and acetonitrile (ACN) were purchased from Aladdin. Stannous chloride dihydrate (SnCl_2_·2H_2_O, ≥ 99.99%) was purchased from Beijing Jinming Biotechnology Co., Ltd. Hydrochloric acid (HCl, 36.0–38.0 wt% in water) was purchased from Fuchen (Tianjin) Chemical Reagent Co., Ltd. N, N-dimethylformamide (DMF, 99.7%) was purchased from Alfa Aesar. 1-methy-2-pyrrolidinone (NMP, 99.5%) was purchased from Beijing InnoChem Science & Technology Co., Ltd. Thioglycolic acid (TGA, > 95.0%) was purchased from TCI America. [6,6]-Phenyl-C61-butyric acid methyl ester (PCBM) was purchased from Jiangsu Sunera Technology Co., Ltd. Chlorobenzene (CB, 99.8%) was purchased from Hebei Bailingwei Super Fine Material Co., Ltd. Ag and Au were purchased from Hebei Rechen New Material Technology Co., Ltd. Carbon electrode paste was purchased from Shanghai MaterWin New Materials Co., Ltd. All chemicals were used as received without any other refinement.

### Device Fabrication

The substrates were cleaned ultrasonically for 20 min in detergent, deionized water, and absolute ethanol, respectively, followed by drying with nitrogen flow. Tin oxide (SnO_2_) was deposited onto FTO/glass substrates by chemical bath deposition (CBD) [32]. The FTO substrates and the CBD solution consisting of 625 mg of urea, 625 μL of HCl, 12.5 μL of TGA, and 137.5 mg of SnCl_2_·2H_2_O per 50 mL of deionized water were loaded onto a glass reaction vessel and kept at a temperature of 90 °C for 4 h, and then washed with deionized water. The obtained substrate was further annealed in an ambient environment at 170 °C for 60 min.

For the perovskite precursor solution, 51.9 mg FAI, 111.3 mg MAI, 484 mg PbI_2_, 10 mg MACl, and 13 mg CsI were dissolved in an NMP/DMF (100 μL/900 μL) mixed solvent. The perovskite precursor solution was spin-coated on top of the ETL at 4000 r min^−1^ for 6 s. The perovskite film was prepared by a low-pressure-assisted method [33, 34]. The film was annealed at 120 °C for 20 min. Finally, the carbon electrode was prepared by blade-coating method and dried at 100 °C for 20 min. The active area of all the devices was 0.09 cm^2^.

For the Li_2_CO_3_-treated device, Li_2_CO_3_ (0.5, 1, 3, or 5 mg) was dissolved in 1 mL of deionized water and then spun on the above FTO/SnO_2_ substrate with UV-ozone treated at 4000 r min^−1^ for 30 s. Subsequently, the film baked on a hot plate at 100 °C for 30 min. The preparation procedures of other layers are the same as described above.

All processes were performed in an ambient atmosphere. All cells were tested unencapsulated.

### Characterizations

The current density–voltage (*J-V*) curves were carried out under simulated AM 1.5G solar irradiation at 100 mW cm^−2^ using a Keithley 2400 semiconductor characterization system with a standard xenon-lamp-based solar simulator (EASISOLAP-50-3A, CROWNTECH, INC.). For the indoor performance tests were measured with indoor light system and HS-IL spectrometer (ILS-30). The light intensity was calibrated using a certified reference cell. Scanning electron microscopy (SEM) images were recorded with a JEOL JSM7610F SEM. The X-ray diffraction (XRD) patterns of the films were measured using a Rigaku SmartLab with Cu K_α_ radiation. The measuring power was 4 kW, and the scanning rate was 8° min^−1^. Photoluminescence (PL) spectra were measured with NanoLog infrared fluorescence spectrometer (Nanolog FL3-2Ihr) using a 450-nm laser as the excitation source. Time-resolved photoluminescence (TRPL) characterization was conducted by an Ultrafast Lifetime Spectrometer (Delta Flex). PL mapping and PL lifetime mapping were measured by ISS Q2 modular confocal microscope. The ultraviolet visible-light (UV–Vis) absorption spectra were collected with a Shimadzu UV-1900 spectrophotometer in the spectral range from 300 to 900 nm. Mott-Schottky measurement was performed using an AMETEK VersaSTAT 3F at frequency of 1 kHz. Electrochemical impedance spectroscopy (EIS) was measured by applying a bias of the open-circuit voltage with an CH1660E electrochemical workstation under dark conditions. The scanning frequency was set between 1 and 105 Hz. Ultraviolet photoelectron spectroscopy (UPS) and X-ray photoelectron spectroscopy (XPS) were measured with ESCALAB 250Xi, and the XPS spectra were calibrated using inorganic carbon 1* s* peak at 284.50 eV as a reference. Atomic force microscopy (AFM), Kelvin probe force microscopy (KPFM) and conductive atomic force microscopy (C-AFM) were conducted using Asylum Research MFP 3D Atomic Force Microscope. Grazing incidence wide-angle X-ray scattering (GIWAXS) measurements were performed using Xenocs Xeuss 2.0 (GI-)SAXS/WAXS/USAXS beamline system with the wavelength of the incident X-ray beam of 0.154 nm. The GIWAXS patterns were collected by a Pilatus 300 K detector with the sample-to-detector distance of 150 mm, and calibrated by the silver behenate standard sample. For the stability test, the unencapsulated c-PSC devices were stored in ambient conditions. ^1^H NMR spectra were measured with a Bruker AVANCE III instrument operating at 400 MHz (FAI or FAI + Li_2_CO_3_ was dissolved in deuterated DMSO to form solution). The IPCE spectra were recorded using an Enli Technology EQE measurement system (QE-R) and the light intensity at each wavelength was calibrated with a standard single crystal Si photovoltaic cell.

All of the measurements were performed in ambient atmosphere at room temperature without any encapsulation.

## Results and Discussion

### Nonradiative Recombination at the Grain Boundaries of HTL-free C-PSCs

**Fig. 1:**
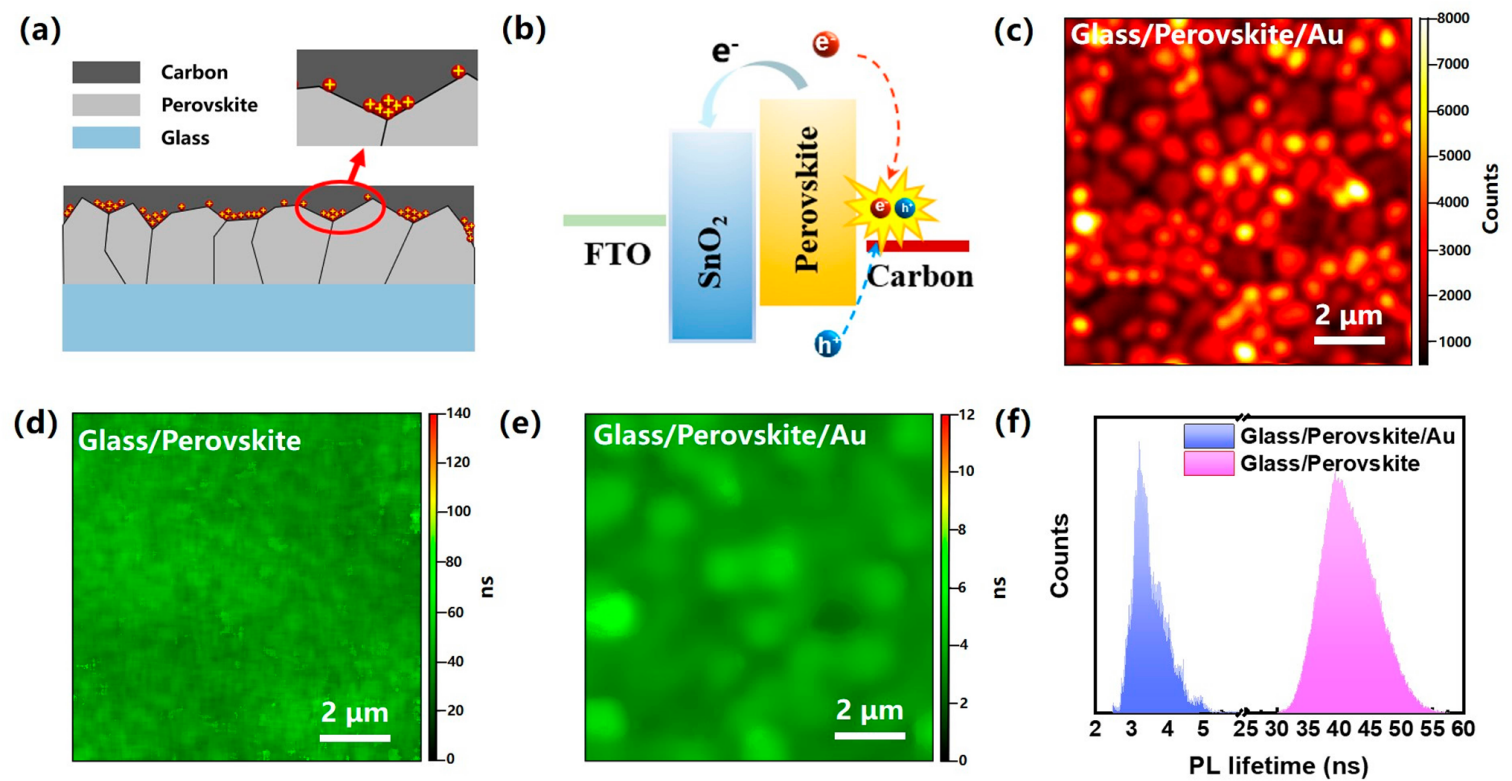
**a** Schematic diagram of the perovskite/carbon interface. **b** Energy-level diagram of the HTL-free C-PSC device. **c** The PL mapping of the glass/perovskite/Au sample. PL lifetime mapping of the **d** glass/perovskite sample and **e** glass/perovskite/Au sample. **f** PL lifetime histogram of glass/perovskite sample and glass/perovskite/Au sample

In a typical HTL-free C-PSC device, the perovskite layer is in direct contact with the highly conductive carbon electrode. In particular, the energy-level mismatch between the commonly used carbon electrodes and the perovskite layers hinders efficient hole extraction at the perovskite/carbon electrode interface [11, 35]. Thus, the defects at the surface and the grain boundaries of the perovskite layer would inevitably facilitate trap-induced nonradiative recombination between the holes and the back transferred electrons at the interface, resulting in significant energy loss, as schematically illustrated in Fig. 1a, b. Time-resolved confocal photoluminescence (PL) microscopy studies were conducted with a 405 nm excitation laser to probe the carrier distribution and lifetime at the perovskite top interface. Since the carbon electrode in the actual C-PSC device is excessively thick to allow sufficient penetration of the incident laser beam as well as collection of the luminescence signal, a thin Au layer of 5 nm is deposited on top of the perovskite layer instead to form the Schottky junction. Figure 1c shows the PL mapping image of the glass/FA_0.3_MA_0.7_PbI_3_/Au sample, where individual grains with the size of hundreds of nanometers to 1 μm in the polycrystalline perovskite thin film are clearly resolved. In addition, we observed a significant anticorrelation between the grain size and the PL intensity aroused from the diffusion-dominated behavior of the carriers and the variation in the carrier population due to different grain sizes, which is well in line with the previous reported confocal imaging studies [36, 37]. The grain boundaries generally exhibit lower luminescence intensity than their interiors, indicating a significant reduction of the carrier density at the grain boundaries. We further examined the PL lifetime of the perovskite thin films with and without Au on the surface as shown in Fig. 1d, e, and the corresponding histogram of the PL lifetime is given in Fig. 1f. It is noteworthy that the lifetime is substantially reduced by one order upon depositing a highly conductive electrode (Au in this case) on the top surface of the perovskite thin film, suggesting faster quenching of charge carriers at the perovskite/electrode interface which is possibly due to the trap-assisted recombination loss at the grain boundaries and surface defects. These observations clearly highlight the primary importance of passivating perovskite/electrode interface to enhance the device performance of the HTL-free C-PSCs.

### Fabrication and Characterization of C-SnO_2_ and Li_2_CO_3_@C-SnO_2_ ETLs

**Fig. 2:**
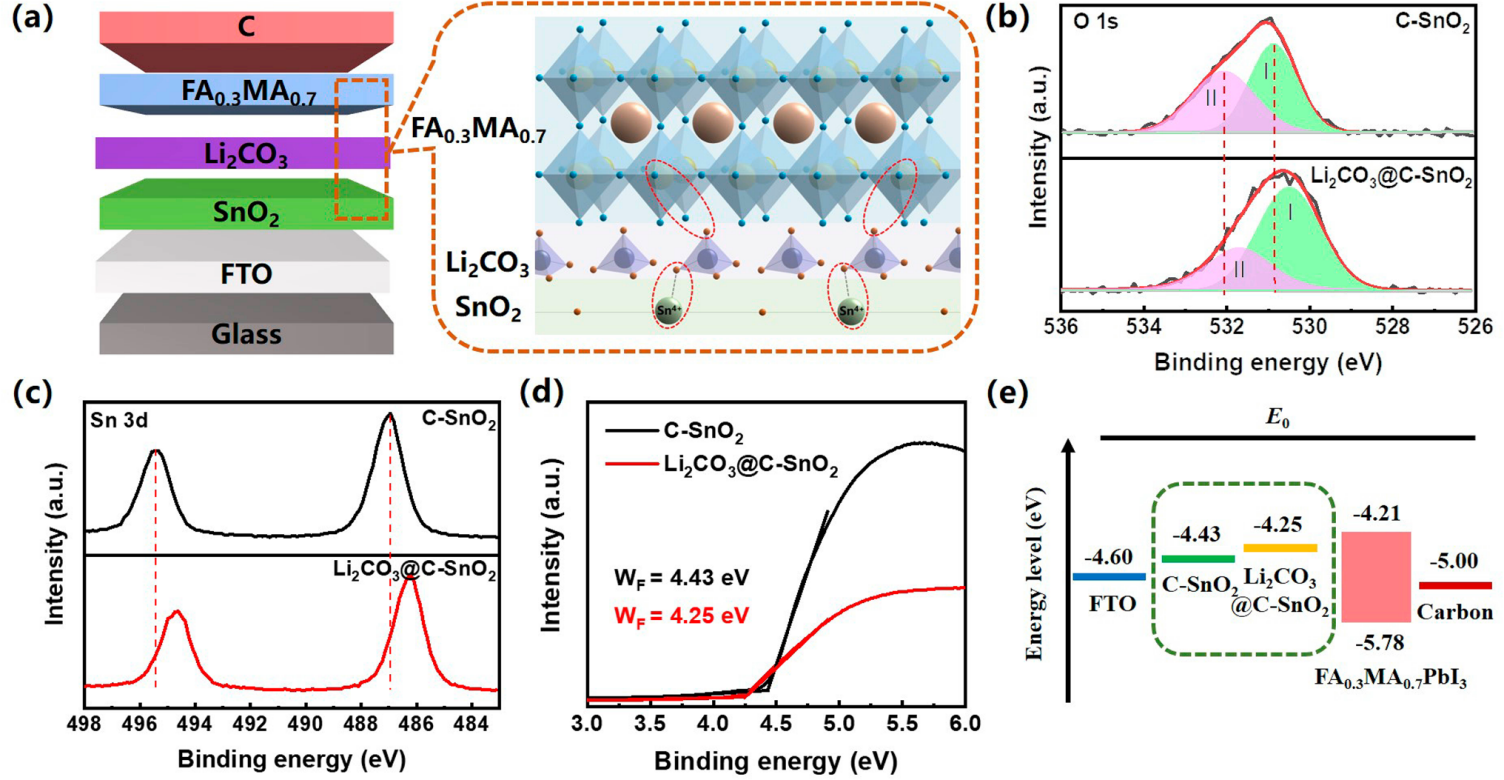
**a** Schematic diagram of device structure and the proposed mechanism of Li_2_CO_3_ modification. XPS spectra of **b** O 1*s* and **c** Sn 3*d* for C-SnO_2_ and Li_2_CO_3_@C-SnO_2_ films. **d** UPS spectra of C-SnO_2_ and Li_2_CO_3_@C-SnO_2_. **e** Energy-level diagram of the C-PSC device

To address the importance of suppressing recombination loss for HTL-free C-PSCs, we employed a multifunctional lithium salt Li_2_CO_3_ at the ETL/perovskite interface, and fabricated HTL-free C-PSC with the architecture of FTO/SnO_2_/Li_2_CO_3_/FA_0.3_MA_0.7_PbI_3_/C (Fig. 2a). SnO_2_-based electron transport layer (ETL) was synthesized on top of the fluorine-doped tin oxide (FTO)/glass substrate by chemical bath deposition following the previously reported procedures [32]. The deposited SnO_2_ is dense and conformal with the underlying FTO layer as shown in Fig. S1a (therefore denoted as C-SnO_2_), and then the C-SnO_2_ surface was further modified with Li_2_CO_3_ (denoted as Li_2_CO_3_@C-SnO_2_) at a concentration of 3 mg mL^−1^ (Fig. S1b). The conductive atomic force microscopy (C-AFM) current images (Fig. S2) indicate increased conductivity of C-SnO_2_ ETL after Li_2_CO_3_ modification, which is corroborated by the dark current–voltage characteristics of the pristine and Li_2_CO_3_-modified SnO_2_ samples as shown in Fig. S3. The enhanced conductivity can be attributed to the diffusion of small-sized Li^+^ ions into the SnO_2_ layer [38, 39]. XPS measurements were performed to study the elemental composition of the pristine and Li_2_CO_3_-modified C-SnO_2_. The obtained spectra were calibrated using the carbon 1*s* peak (284.8 eV) as the reference position (Fig. S4). Figure 2b shows the O 1*s* spectra for C-SnO_2_ and Li_2_CO_3_@C-SnO_2_ samples, which can be deconvoluted into two Gaussian components centered at 531.5 and 532.7 eV, respectively. The peak centered at 531.5 eV (denoted as Peak I) is generally assigned to the lattice oxygen, while the peak at the higher energy side (denoted as Peak II) is usually attributed to the loosely bound chemisorbed oxygen O_2_^−^ at the sample surface [38, 40]. Compared to the O 1*s* spectrum of the pristine C-SnO_2_ sample, Peak I of Li_2_CO_3_@C-SnO_2_ sample is shifted to a lower binding energy of 531.1 eV with considerably enhanced relative intensity, whereas the relative intensity of Peak II is reduced. It has been previously reported that during the annealing process of SnO_2_ in ambient environment, physically adsorbed O_2_ molecules tend to transfer to chemisorbed O_2_^−^ at the defect sites on the SnO_2_ surface [41]. Therefore, the reduced relative intensity of Peak II could be attributed to the reduced defect sites at SnO_2_ surface, which are likely passivated by Li_2_CO_3_. In addition, the characteristic peaks in the O 1*s* and Sn 3*d* spectra are shifted toward lower binding energy after Li_2_CO_3_ modification as shown in Fig. 2b, c, suggesting the presence of electronegative anion components (CO_3_^2−^ in this case) on SnO_2_ surface [38, 42].

UPS measurements were performed to evaluate the energy level of C-SnO_2_ and Li_2_CO_3_@C-SnO_2_, as shown in Fig. 2d. The work function (denoted as W_F_) values of C-SnO_2_ and Li_2_CO_3_@C-SnO_2_ are 4.46 and 4.25 eV, respectively. Combining the energy level of the SnO_2_ ETLs and FA_0.3_MA_0.7_PbI_3_ perovskite layer obtained by UPS and UV–visible absorption spectroscopy (Fig. S5), the energy diagram of devices incorporating C-SnO_2_ and Li_2_CO_3_@C-SnO_2_ as ETLs is depicted in Fig. 2e. An optimized energy-level alignment is observed for the Li_2_CO_3_@C-SnO_2_/perovskite interface, which is expected to facilitate electron extraction and transport of photogenerated carriers. The improved conductivity, reduced defect sites, and optimized energy alignment of SnO_2_ ETL triggered by Li_2_CO_3_ modification would potentially facilitate charge extraction and transport at the ETL/perovskite interface, in accord with previous reports employing lithium salts as dopants or surface modifiers for metal oxide ETL [38].

### Characterization of the Perovskites on C-SnO_2_ and Li_2_CO_3_@C-SnO_2_

**Fig. 3:**
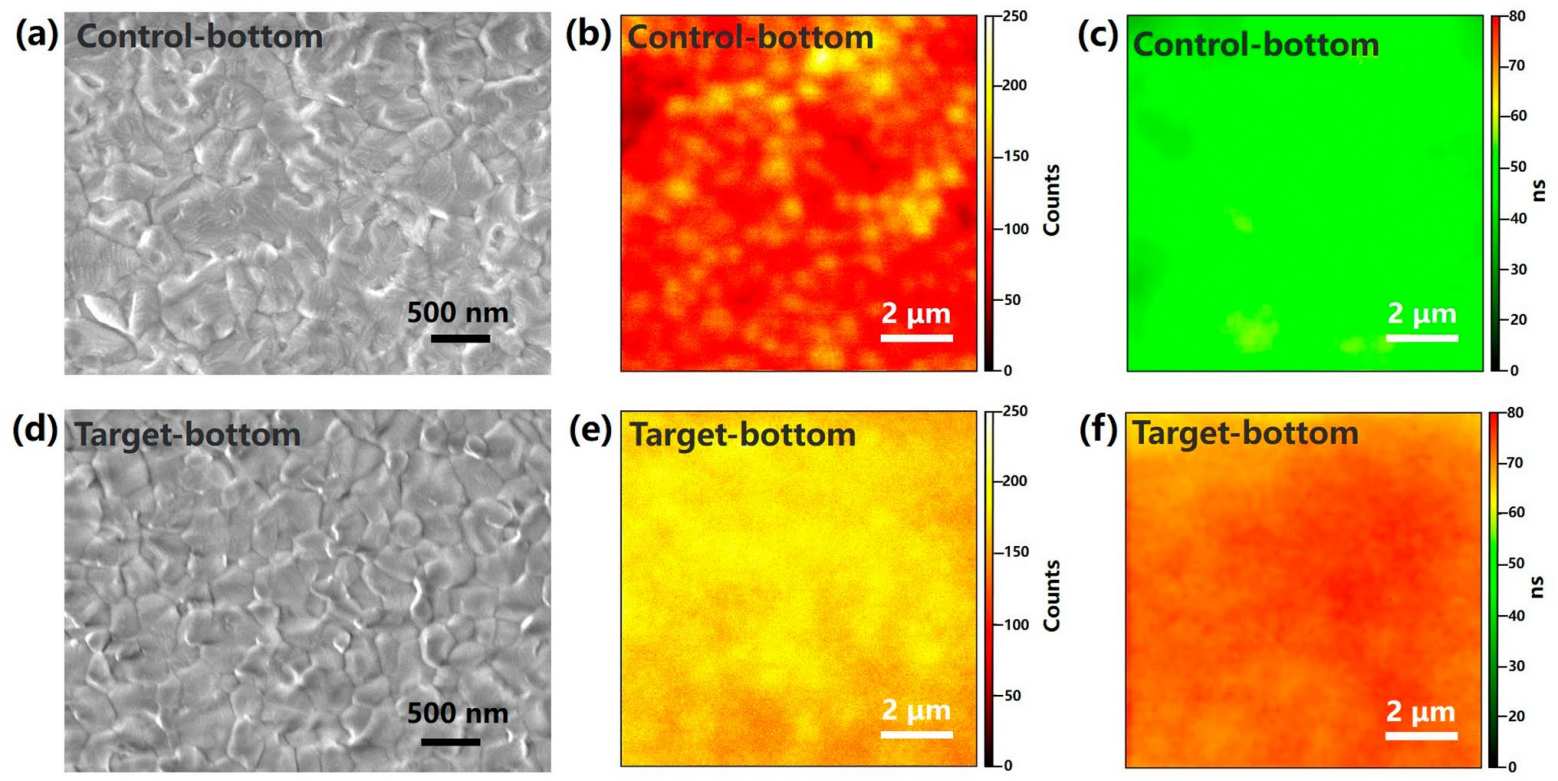
The bottom interface of control sample. **a** SEM image, **b** PL mapping, **c** PL lifetime mapping. The bottom interface of target sample. **d** SEM image, **e** PL mapping, **f** PL lifetime mapping

Aside from the influences on the ETL properties, we continued to investigate the impact of lithium salt modification on the perovskite absorber layer starting from its bottom interface. The perovskite layers deposited on pristine and Li_2_CO_3_-modified SnO_2_ ETLs were peeled off from the substrates with the aid of a UV-curable epoxy resin and then subjected to further characterizations. From the SEM images of the perovskite bottom interface as shown in Fig. 3a, d, reduced grain sizes are observed for the perovskite film deposited on the Li_2_CO_3_@C-SnO_2_ (the target sample) compared with the sample prepared on the pristine C-SnO_2_ (the control sample). The remarkably enhanced PL intensity and lifetime of the target sample over the control sample revealed by the time-resolved confocal PL mapping studies (Figs. 3b, c, e, f, and S6) indicates suppressed recombination loss by Li_2_CO_3_ modification. In addition, the strong interaction of CO_3_^2−^ with FA^+^ is evidenced by the nuclear magnetic resonance (NMR) study as shown in Fig. S7, which enables passivation of FA related defects with the presence of Li_2_CO_3_ at the perovskite bottom interface [38].

**Fig. 4:**
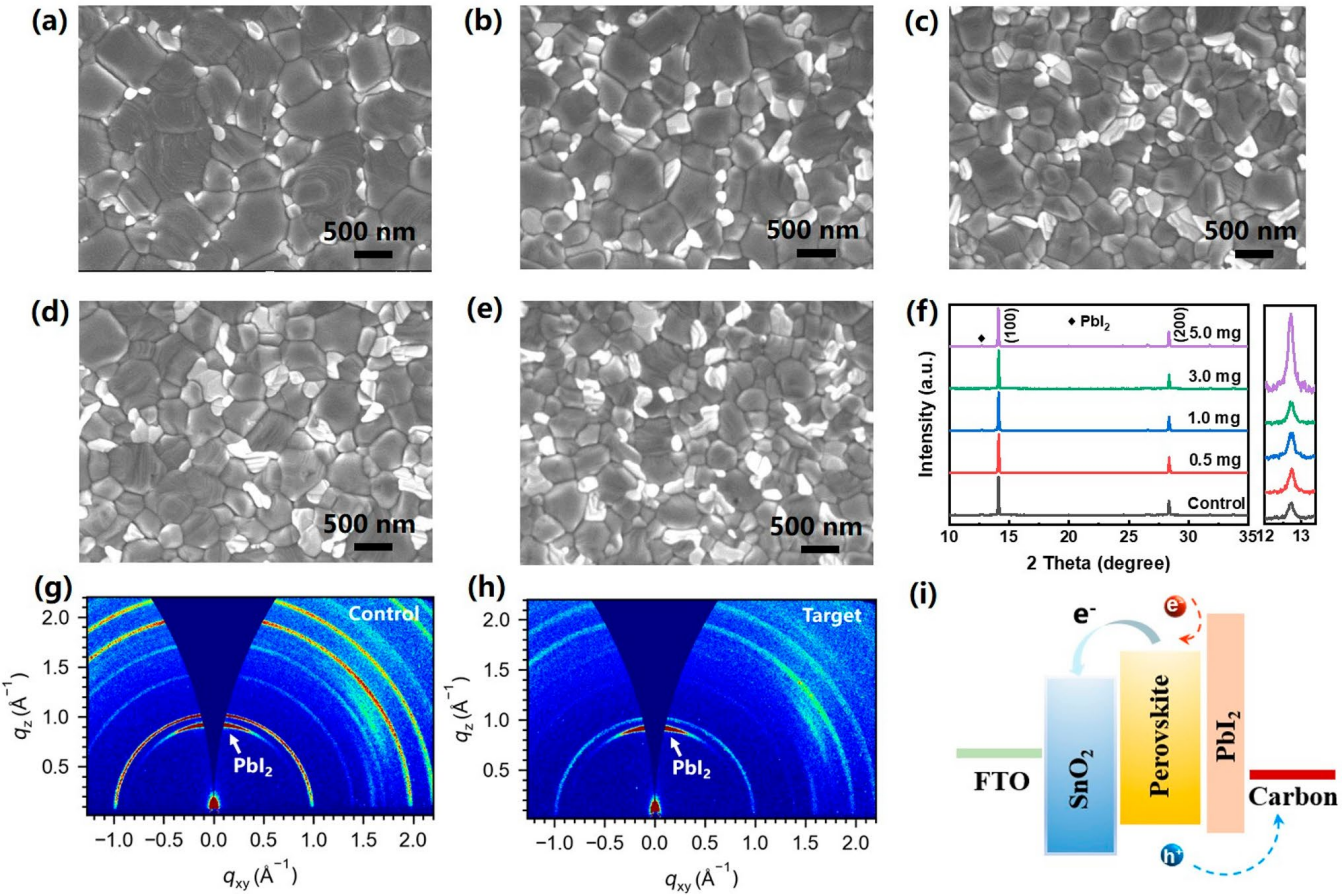
SEM top-view images of perovskites on C-SnO_2_ substrates with various Li_2_CO_3_ modification conditions **a** pristine C-SnO_2_, **b** 0.5 mg mL^−1^, **c** 1.0 mg mL^−1^, **d** 3 mg mL^−1^, **e** 5 mg mL^−1^. **f** XRD patterns of the perovskite films on the modified SnO_2_ with various concentrations of Li_2_CO_3_. GIWAXS patterns of the perovskite films deposited on **g** C-SnO_2_ and **h** Li_2_CO_3_@C-SnO_2_. **i** Schematic energy band diagram

We then examined the effect of Li_2_CO_3_ modification on the top surface of the perovskite absorber layer. The morphology of the FA_0.3_MA_0.7_PbI_3_ films deposited on the pristine and modified ETLs was characterized using a scanning electron microscope. From the top-view SEM images of the obtained perovskite films as shown in Fig. 4a–e and the corresponding histograms of grain size distribution (Fig. S8), it is clearly observed that the average grain size of the perovskite films reduces as the Li_2_CO_3_ concentration increases (0, 0.5, 1, 3, and 5 mg mL^−1^, respectively). Also noteworthy is the unambiguous correlation between the Li_2_CO_3_ modification concentration and the presence of “white” crystalline particulates at the grain boundaries. The XRD patterns of the obtained perovskite films are shown in Fig. 4f, where the diffraction peak at 2*θ* ≈ 12.7° corresponding to lead iodide (PbI_2_) slightly rises with increasing Li_2_CO_3_ concentration (Fig. 4f, right panel). The presence of increased PbI_2_ content at the perovskite top surface is further evidenced by grazing incidence wide-angle X-ray scattering (GIWAXS) characterization, as shown in Fig. 4g, h. Based on the combined morphology and structure characterizations, we assign the “white” crystallites as PbI_2_. By adopting Li_2_CO_3_ modification, the crystalline PbI_2_ segregated at the grain boundaries of the perovskite top surface can passivate the grain boundary defects, thus leading to the suppression of nonradiative recombination (Fig. 4i) [43]. In addition, the more insulating PbI_2_ domains can effectively block the undesired electron back transfer from the perovskite to the carbon electrode, thus benefiting the overall photovoltaic performance [44].

To further elucidate the origin of the morphology and compositional changes observed at the perovskite top surface, particularly the formation of PbI_2_ at the grain boundaries upon Li_2_CO_3_ modification, we probed the correlation between the perovskite compositions and the PbI_2_ crystallite formation by depositing perovskite films with various cation compositions on the pristine and Li_2_CO_3_-modified SnO_2_ ETLs. When deposited on pristine SnO_2_ ETLs, pure FAPbI_3_ and FA_0.3_MA_0.7_PbI_3_ films exhibit large grains of ~ 1 μm, while pure MAPbI_3_ film shows distinctly smaller grains of ~ 100–300 nm (Fig. S9a, c, e). In addition, negligible PbI_2_ is observed in the pure MAPbI_3_ film compared with the FA-containing perovskite films. Once the perovskite films were deposited upon the SnO_2_ ETLs modified with saturated Li_2_CO_3_ aqueous solution (corresponding to a Li_2_CO_3_ concentration of 8 mg mL^−1^), the presence of PbI_2_ were merely changed in the pure FAPbI_3_ film, but tremendously increased in the MA-containing perovskite films (Fig. S9b, d). This is also confirmed by the XRD patterns of the corresponding perovskite films as shown in Fig. S10. The drastically changed top surface morphology of MA-containing perovskite films inevitably evidences a more significant role of MA cation in the Li_2_CO_3_-induced PbI_2_ formation than FA cation.

In view of the experimental observation discussed above, we speculate that the formation of PbI_2_ crystallites is facilitated by the thermally assisted interaction of Li_2_CO_3_ and MA-containing perovskites near the ETL/perovskite interface. To identify the possible chemical reaction between Li_2_CO_3_ and the MA-containing species, we deposited MAPbI_3_ films on pristine and Li_2_CO_3_-modified substrates followed by annealing at 110 °C for 20 min. The perovskite films were then peeled off from the substrates for XRD characterization of their bottom interfaces. From the resulting XRD patterns shown in Fig. S11, discernible peaks at 25.69° corresponding to the (111) reflections of LiI can be observed, along with enhanced peak intensity corresponding to PbI_2_, which represents direct evidence of LiI as one of the reaction products of Li_2_CO_3_ and MA-containing species. To further identify the possible reaction pathway, we examined the product of solid-state reaction of MAI with Li_2_CO_3_ in humid air condition using powder XRD, where the characteristic diffraction peaks of LiI**·**3H_2_O are clearly spotted as shown in Fig. S12. It has been reported that MAPbI_3_ can reversibly decompose into methylamine (CH_3_NH_2_), hydrogen iodide (HI), and solid PbI_2_ remnant under thermal stress [45]. Considering the spontaneous reaction between Li_2_CO_3_ and HI, we propose the following reaction pathway leading to the presence of PbI_2_ crystallites in the MA-containing perovskites:

1 $${\text{CH}}_{{3}} {\text{NH}}_{{3}} {\text{I}}\mathop \rightleftharpoons \limits^{\Delta } {\text{CH}}_{{3}} {\text{NH}}_{{2}} + {\text{HI}}$$

2 $${\text{Li}}_{{2}} {\text{CO}}_{{3}} + 2{\text{HI}}\mathop{\longrightarrow}\limits^{\Delta }2{\text{LiI}} + {\text{CO}}_{{2}} + {\text{H}}_{{2}} {\text{O}}$$

For the MA-containing perovskite wet films annealed at elevated temperature, the thermally promoted reversible decomposition of MAI is most likely to occur near the substrate, forming gaseous intermediate products CH_3_NH_2_ and HI. While CH_3_NH_2_ is potentially released upon annealing, HI is subsequently reacted with Li_2_CO_3_ at the ETL/perovskite interface, resulting in solid phase LiI near the bottom interface of the perovskite layer. Other reaction products according to Eq. (2) include CO_2_ and H_2_O, both of which are expected to be thoroughly removed from the perovskite film with extended annealing time. The loss of MAI via the proposed pathway would eventually lead to the increased presence of PbI_2_ crystallites in the resulting perovskite film. Interestingly, grazing incidence XRD (GIXRD) characterization suggests a vertical distribution gradient of PbI_2_ within the perovskite film that higher PbI_2_ content is observed the at the top surface compared to the bottom interface (Fig. S13, Table S1). Such vertical distribution gradient would probably be linked to the segregation of PbI_2_ [43].

We also performed the atomic force microscopy (AFM) for the perovskite films deposited on C-SnO_2_ and Li_2_CO_3_@C-SnO_2_ at a Li_2_CO_3_ concentration of 3 mg mL^−1^ (namely control sample and target sample, respectively). The AFM images (Fig. S14) show the top surface morphology of the perovskite films in a 5 × 5 µm^2^ scan range, and the corresponding root mean-square averages (*R*_q_) of height deviation are 32.19 and 29.64 nm for the control and the target samples, respectively. The significantly reduced *R*_q_ of the target sample implies a considerably flatter top surface, which is favorable for the contact between the perovskite and the carbon electrode.

### Photovoltaic Performance of Li_2_CO_3_@C-SnO_2_-based C-PSCs

**Fig. 5:**
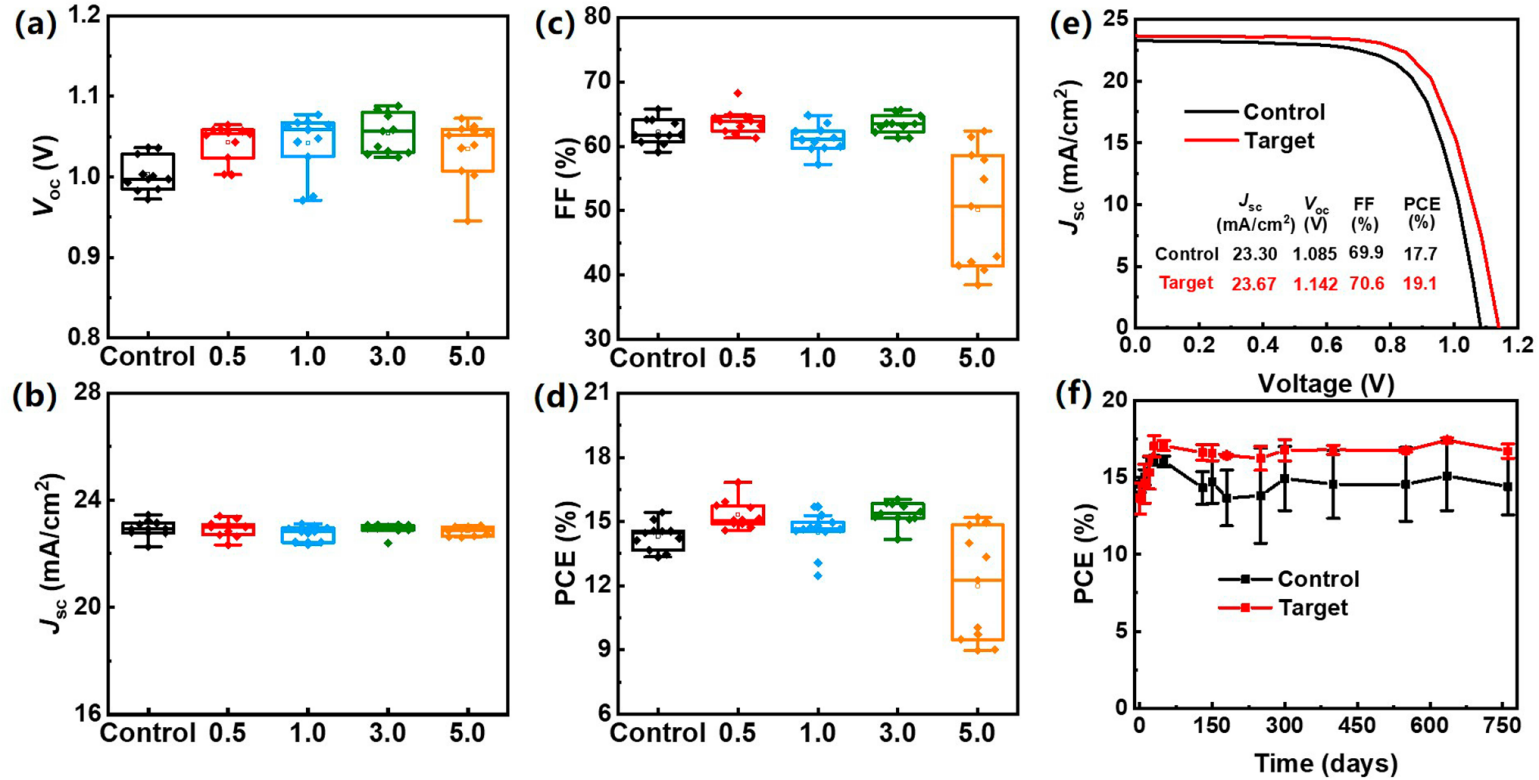
Photovoltaic parameters of perovskite solar cells based on SnO_2_ with various Li_2_CO_3_ modification conditions **a** *V*_oc_, **b** *J*_sc_, **c** FF, and **d** PCE. **e** *J *–*V* curves of the best-performing control and target devices with reverse scan. **f** PCE of control and target devices tracked under ambient condition (temperature ~ 15–30 °C, relative humidity ~ 20%) without any encapsulation

Based on the above discussion, Li_2_CO_3_ modification of SnO_2_ ETL shows versatility in passivating both perovskite top and bottom interfaces. As for the bottom interface, reduced defects in both SnO_2_ ETL and perovskite layer are achieved, along with increased ETL conductivity and enhanced energy alignment of the ETL/perovskite interface. Regarding the perovskite top surface, nonradiative recombination loss is suppressed by Li_2_CO_3_-induced formation of PbI_2_ at grain boundaries. To investigate the effect of the Li_2_CO_3_ modification on device performance, we fabricated C-PSCs based on SnO_2_ ETL modified with various Li_2_CO_3_ concentrations. Negligible changes in the absorption edges were observed for the perovskite films deposited on SnO_2_ ETL with different Li_2_CO_3_ modification concentrations, as shown in Fig. S15. The device performance of the C-PSCs with various Li_2_CO_3_ modification conditions was then assessed, and statistical performance parameters including *V*_oc_, short-circuit current (*J*_sc_), fill factor (FF), and PCE from 11 individual devices for each Li_2_CO_3_ concentration are summarized in Fig. 5a–d and Table S2. Among all the Li_2_CO_3_ modification concentrations, devices with 3 mg mL^−1^ of Li_2_CO_3_ modification exhibit enhancement for all the photovoltaic parameters. Therefore, 3 mg mL^−1^ was defined as the optimal concentration for Li_2_CO_3_ modification. We also fabricated Li_2_CO_3_-modified C-PSC devices with various perovskite precursor concentrations to determine the optimal value of 1.4 M (Fig. S16 and Table S3). The C-PSCs based on pristine and Li_2_CO_3_-modified C-SnO_2_ ETLs with optimal concentration are referred to as the control device and target device, respectively, in the later discussion. Each group of 20 individual devices was prepared under the same experimental conditions, and their photovoltaic parameters were statistically analyzed, as shown in Fig. S17. The control devices exhibited an average PCE of 16.3%, with a *V*_oc_ of 1.075 V, a *J*_sc_ of 23.31 mA cm^−2^, and a FF of 65.2%. In contrast, the target devices demonstrated a significant improvement, with the average *V*_oc_ and FF increasing to 1.136 V and 69.2%, respectively. This enhancement resulted in a notable rise in the average PCE to 18.5%. The best-performing control device showed a PCE of 17.7%, combined with a *V*_oc_ of 1.085 V, a *J*_sc_ of 23.30 mA cm^–2^, and an FF of 69.9%. The target device exhibited considerably improved photovoltaic performance, with the champion device reaching a PCE of 19.1% (corresponding to a *V*_oc_ of 1.142 V, a *J*_sc_ of 23.67 mA cm^−2^, and an FF of 70.6%). The performance enhancement is mainly attributed to the *V*_oc_ increased from 1.085 to 1.142 V, demonstrating the importance of reducing photon energy loss for solar cells [46]. It was worth noting that the *V*_oc_ of our HTL-free C-PSCs is comparable to the metal electrode-based devices with HTL (Fig. S18 and Table S4). Besides, the *J*_sc_ increment for the control and target devices are in good agreement with the incident photon-to-current efficiency (IPCE) measurements (Fig. S19). The steady output of target device is significantly higher than that of control device (Fig. S20). We have fabricated PSC with Li_2_CO_3_-coated conventional SnO_2_ (spin casting the colloidal dispersion) as ETL, and its PCE (15.0%, Fig. S21 and Table S5) is lower than that of target device (19.1%), indicating conformal SnO_2_ is indeed very important for enhancing the performance of HTL-free C-PSCs. We further tested the long-term stability of the unencapsulated control and target devices in ambient conditions (temperature ~ 15–30 °C, relative humidity ~ 30%), as shown in Figs. 5f and S22. Interestingly, the PCE of both control and target devices gradually increased and reached the maximum after storage in ambient conditions for 30 days. Upon prolonged ambient storage, while the control device showed a slow decrease in PCE and FF, a negligible reduction is observed for all the parameters of the target device, demonstrating excellent long-term stability. For the operational stability test, C-PSC device was tracked at maximum power point (MPP) under continuous 1 sun illumination, as shown in Fig. S23. The cell maintains ~ 90% of the initial efficiency after 360 h continuous operation. In consistency with the enhanced device stability, the perovskite film also exhibits superior stability with Li_2_CO_3_ modification, as evidenced by the virtually identical crystalline and morphology characteristics after storage in ambient conditions for 30 days (Fig. S24).

### Photovoltaic Performance under Weak Light Intensity Conditions

**Fig. 6:**
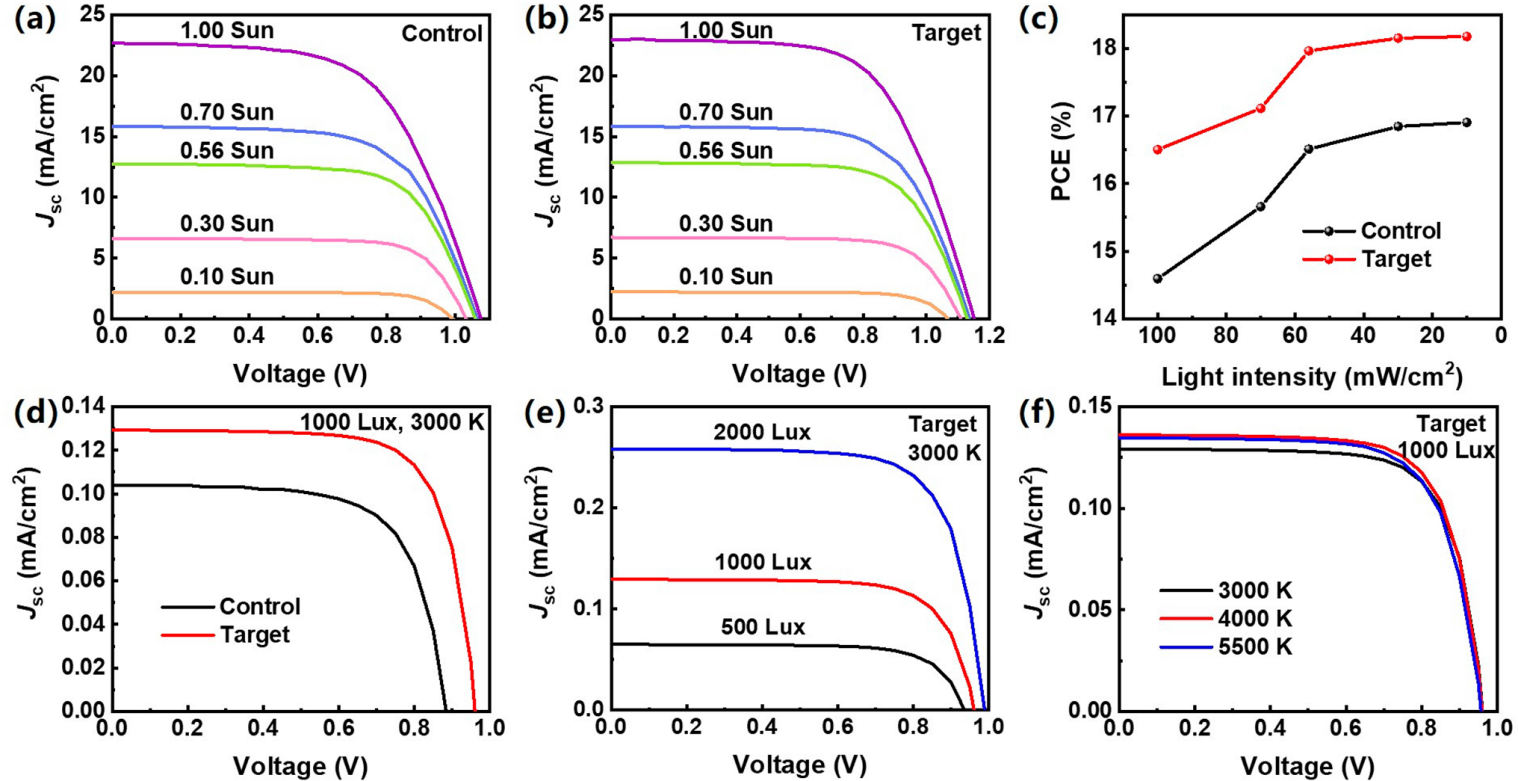
*J–V* curves of the C-PSCs measured under simulated solar illumination at various light intensity conditions **a** control device, **b** target device. **c** Light intensity dependent PCE characteristics of the C-PSCs under simulated solar illumination. **d** *J *–*V* curves of the devices under 3000 K LED illumination at 1000 lx. **e** *J *–*V* curves of the target under 3000 K LED illumination with various light intensities. **f** *J *–*V* curves of the target under LED illumination with fixed light intensity (1000 lx) and various color temperatures

We examined the photovoltaic performance of the C-PSCs under weak illumination conditions. The *J *–*V* characteristics of the control and target devices under simulated solar (AM1.5G) illumination with various light intensities are shown in Fig. 6a, b. Both devices exhibit increased power conversion efficiencies with reduced light intensity, as shown in Fig. 6c. To further extend our knowledge on the weak light behavior of the C-PSCs, we tested the device performance of control and target devices under LED illumination with various luminous intensities and color temperatures (CTs). Figure S25 shows the spectra of LED illumination at different color temperatures. When measured under the LED illumination at 1000 lx with a CT of 3000 K, the target device achieved an efficiency as high as 32.4%, while the control device has a conversion efficiency of 22.4% (Fig. 6d), with the detailed device parameters summarized in Table S6. Figure 6e shows the performance dependence of the target device on the intensity of the LED illumination with CT = 3000 K, with the detailed device parameters summarized in Table S7. The PCE of the target device exceeds 30% in all illumination conditions tested, and specifically, we observed the maximum efficiency of 33.2% for the target device under LED illumination at 2000 lx and 3000 K, as shown in Figs. 6f and S26, Tables S8 and S9. This efficiency ranks among the highest reported for low-energy harvesting C-PSCs to date (Table S10) [47–50]. These results demonstrate the excellent weak light photovoltaic performance of the HTL-free C-PSCs based on Li_2_CO_3_-modified SnO_2_ ETL.

### Mechanism for the Improved Performance

**Fig. 7:**
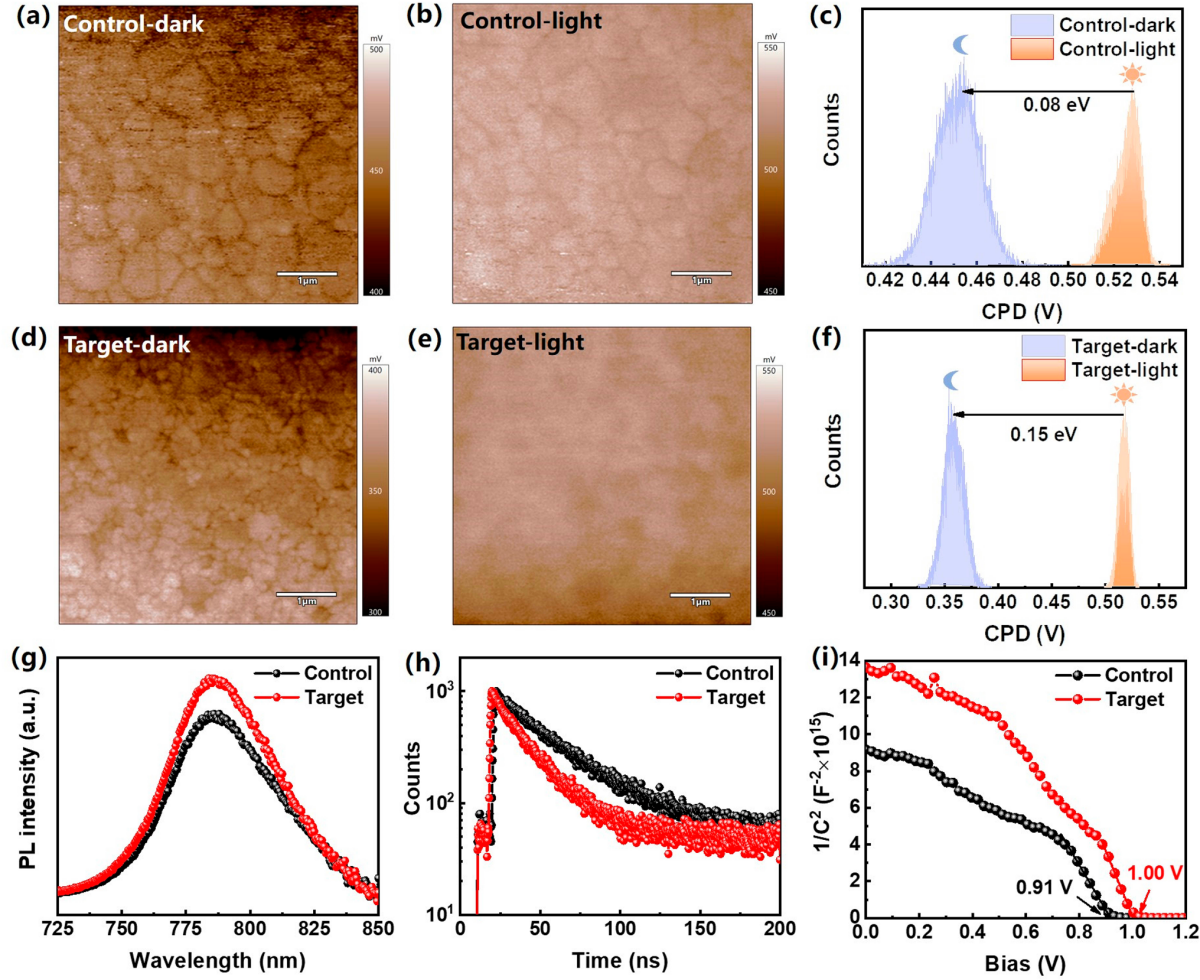
KPFM images for perovskite film on C-SnO_2_: **a** under the dark condition, **b** under the light condition, and **c** CPD. KPFM images for perovskite film on Li_2_CO_3_@C-SnO_2_: **d** under the dark condition, **e** under light condition, and **f** CPD. **g** Steady-state PL spectra of the control and target samples. **h** TRPL spectra of the control and target samples. **i** 1/C^2^ versus applied voltage plots (Mott-Schottky) of control and target devices

Charge separation at the ETL/perovskite interface was evaluated by Kelvin probe force microscopy (KPFM) as shown in Fig. 7a–f. The difference between the average contact potential difference (CPD) of obtained with and without light illumination is 0.08 eV for the control sample, while this value substantially increased to 0.15 eV for the target sample (Fig. 7c, f). The variations of surface potentials obtained under illumination or in dark conditions could reflect the nature of carrier dynamics within perovskite films [51]. Larger potential difference implies that the photogenerated carriers in the perovskite layer are more efficiently separated at ETL/perovskite interface. The charge extraction and transfer properties at ETL/perovskite interface were further evaluated by measuring the steady-state photoluminescence and time-resolved photoluminescence (TRPL) spectra, as shown in Fig. 7g, h. The PL intensity of the target sample is enhanced compared to that of the control sample, suggesting effective suppression of the interface defects of the perovskite film by Li_2_CO_3_ modification. In addition, the target sample shows a shorter PL lifetime compared with that of the control film, indicating more efficient carrier extraction lead by Li_2_CO_3_ modification. To quantitatively evaluate the trap density of the perovskite films, we performed space charge limited current (SCLC) measurement by fabricating electron-only devices with the configuration of FTO/C-SnO_2_/perovskite/PCBM/Ag and FTO/Li_2_CO_3_@C-SnO_2_/perovskite/PCBM/Ag, as shown in Fig. S27. The log–log plot of the current–voltage curve consists of three regimes with the slope *n* = 1, *n* > 3, and *n* = 2, namely ohmic, trap-filled limited (TFL), and Child’s region, respectively. The defect density *n*_t_ was calculated according to Eq. (3):

3 $$V_{{{\text{TFL}}}} = \frac{{en_{t} L^{2} }}{{2\varepsilon_{0} \varepsilon_{r} }}$$

where *ε*_0_, *ε*_r_, *e*, *L* are the vacuum permittivity (8.854 × 10^−14^ F cm^−1^), the relative dielectric constant of perovskite film, the elementary charge (1.6 × 10^−19^ C), and the thickness of the perovskite layer, respectively [52]. The trap-filled limit voltage (*V*_TFL_) is chosen as the crossing point between the ohmic regime tangent and the trap-filled-limited regime tangent, and the corresponding the *V*_TFL_ of the control and target devices were 0.15 and 0.13 V, respectively, indicating lower defect density of the target sample with Li_2_CO_3_ modification. We also fabricated hole-only devices with the configuration of FTO/PTAA/perovskite/Spiro-OMeTAD/Au and FTO/PTAA/Li_2_CO_3_/perovskite/Spiro-OMeTAD/Au to examine the effect of Li_2_CO_3_ modification on hole defect states as shown in Fig. S28. The corresponding *V*_TFL_ of the control and target devices were 0.40 and 0.18 V, respectively, indicating strong passivation effect of Li_2_CO_3_ modification for hole defect states. We measured the *J *–*V* curves of the control and target devices under dark conditions as shown in Fig. S29, where a lower dark saturation *J*_sc_ was observed in the target device as compared with the control device, indicating the suppressed leakage current with Li_2_CO_3_ modification. The carrier transport and recombination behaviors were evaluated using electrochemical impedance spectroscopy (EIS) [42, 53]. Figure S30 shows the Nyquist plots of the control and target devices with a bias voltage of 0.4 V under a dark condition. The larger semicircle diameter of target device suggests increased *R*_rec_ in comparison to the control sample, which implies that Li_2_CO_3_@C-SnO_2_ ETL is more effective in suppressing charge recombination processes. In addition, we conducted Mott-Schottky measurements to examine the built-in potential of the control and target devices. The relationship between capacitance and potential is given by Eq. (4):

4 $$\frac{1}{{C^{2} }} = \frac{2}{{qA^{2} \varepsilon_{r} \varepsilon_{0} N}}(V_{{{\text{bi}}}} - V)$$

where *C* is the measured capacitance, *q* is the elementary charge, *A* is the active area of the device, *ε*_r_ is the relative dielectric constant of the perovskite layer, *ε*_0_ is the permittivity of free space,* N* is the doping concentration of perovskite film, and *V* is the applied bias [13, 54]. The *V*_bi_ of the target device was 1.00 V, which is considerably higher than 0.91 V of the control device (Fig. 7i). The enhanced built-in potential (*V*_bi_) can generate a stronger driving force for the separation of photogenerated carriers and immensely suppress recombination at the interfaces of the perovskite layer and the neighboring charge transport layers.

## Conclusions

In summary, to address the issue of significant *V*_oc_ loss in HTL-free carbon-based PSCs, we present an effective and feasible approach for dual interfacial modification of the perovskite layer by introducing Li_2_CO_3_ at the ETL/perovskite interface. The PCE of HTL-free C-PSCs was boosted from 17.7 to 19.1%, with an eminently improved* V*_oc_ reaching 1.142 V. The enhanced photovoltaic performance is primarily assigned to the effectively suppressed *V*_oc_ loss, which is further linked to the defect passivation and enhanced carrier transport/extraction at the perovskite top and bottom interfaces with Li_2_CO_3_ modification. More importantly, the Li_2_CO_3_@SnO_2_-based device achieved a PCE of 33.2% under LED illumination at 2000 lx with a color temperature of 3000 K, which is one of the highest efficiencies for HTL-free C-PSCs under low light under low-intensity illumination conditions. This work provides a simple and feasible strategy to fabricate low-cost, high-efficient perovskite solar cells for low-energy harvesting applications.

## Supplementary Information

Below is the link to the electronic supplementary material.

Supplementary file1 (DOCX 12157 KB)
