# Supplementary material for: Reducing the Voc Loss of Hole Transport Layer-Free Carbon-Based Perovskite Solar Cells via Dual Interfacial Passivation
Source: Nanomicro Lett. 2025 May 19;17:258. doi: 10.1007/s40820-025-01775-4 (PMC12089553; doi:10.1007/s40820-025-01775-4)
Supplement: Supplementary file 1 — Supplementary file1 (DOCX 12157 KB) [file 40820_2025_1775_MOESM1_ESM.docx]

Supporting Information for

**Reducing the *V*_oc_ Loss of Hole Transport Layer Free Carbon-Based Perovskite Solar Cells via Dual Interfacial Passivation**

Xian Zhang^1^, Fangzhou Liu^1^, Yan Guan^2^, Yu Zou^3^, Cuncun Wu^1,^ *, Dongchang Shi^1^, Hongkai Zhang^1^, Wenjin Yu^3^, Dechun Zou^2^, Yangyang Zhang^1,^ *, Lixin Xiao^3,^ *, and Shijian Zheng^1,^ *

^1^ Key Laboratory of Materials Laminating Fabrication and Interface Control Technology of Tianjin, School of Materials Science and Engineering, Hebei University of Technology, Tianjin 300401, P. R. China

^2^ College of Chemistry and Molecular Engineering, Peking University, Beijing 100871, P. R. China

^3^ State Key Laboratory for Mesoscopic Physics and Department of Physics, Peking University, Beijing 100871, P. R. China

*Corresponding authors. E-mail: [cuncunwu@hebut.edu.cn](mailto:cuncunwu@hebut.edu.cn) (Cuncun Wu); [yyzhang@hebut.edu.cn](mailto:yyzhang@hebut.edu.cn) (Yangyang Zhang); [lxxiao@pku.edu.cn](mailto:lxxiao@pku.edu.cn) (Lixin Xiao); [sjzheng@hebut.edu.cn](mailto:sjzheng@hebut.edu.cn) (Shijian Zheng)

**Supplementary Figures and Tables**


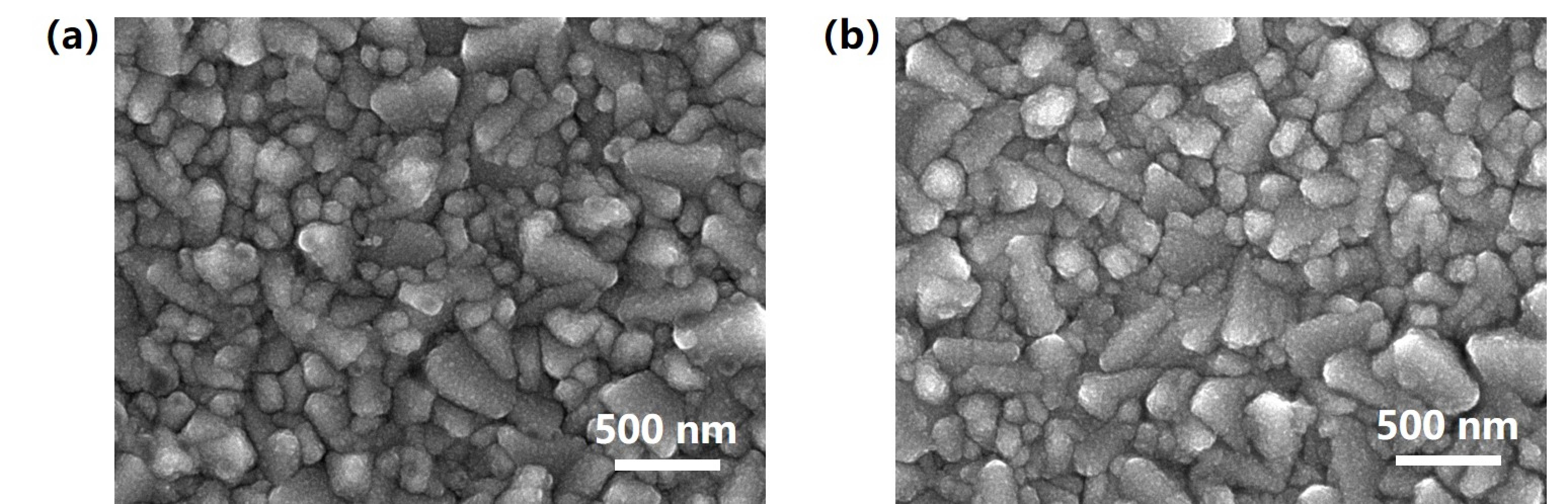


**Fig. S1** SEM images of **(a)** C-SnO_2_ and **(b)** Li_2_CO_3_@C-SnO_2_


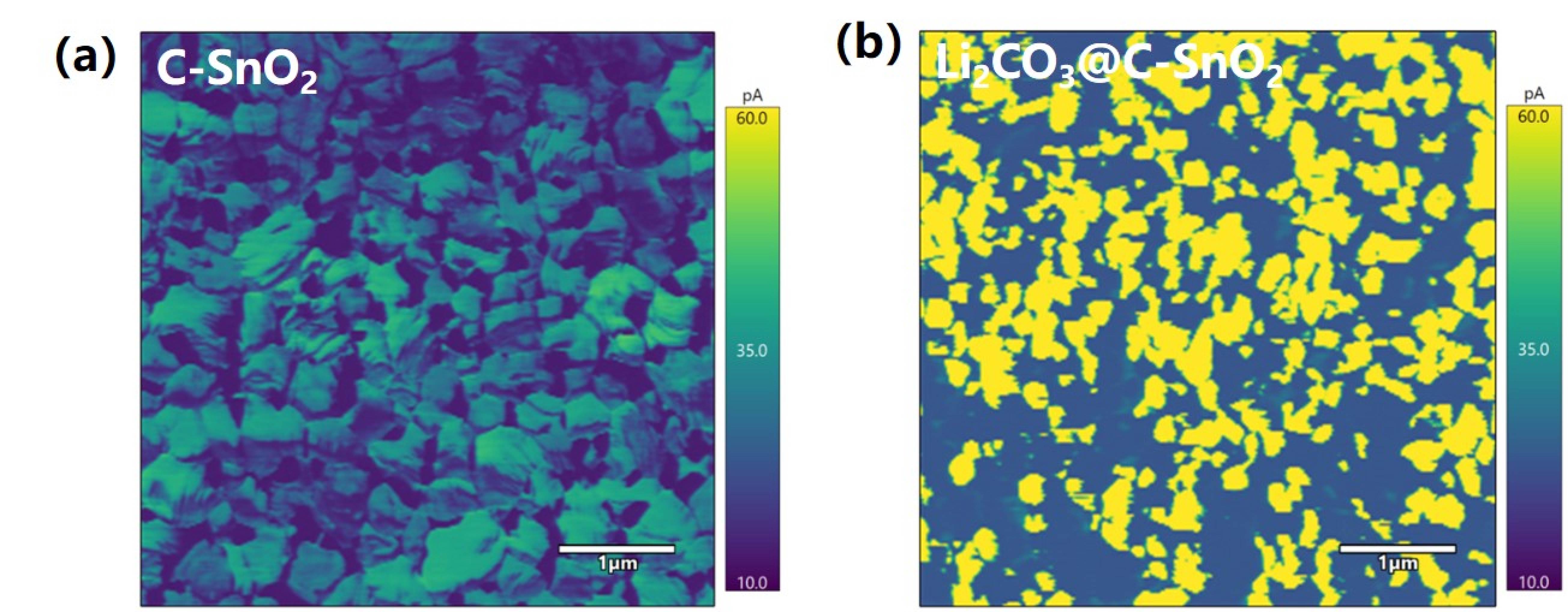


**Fig. S2** C-AFM images of **(a)** C-SnO_2_, **(b)** Li_2_CO_3_@C-SnO_2_. A bias of +200 mV is applied between the conductive probe and the sample


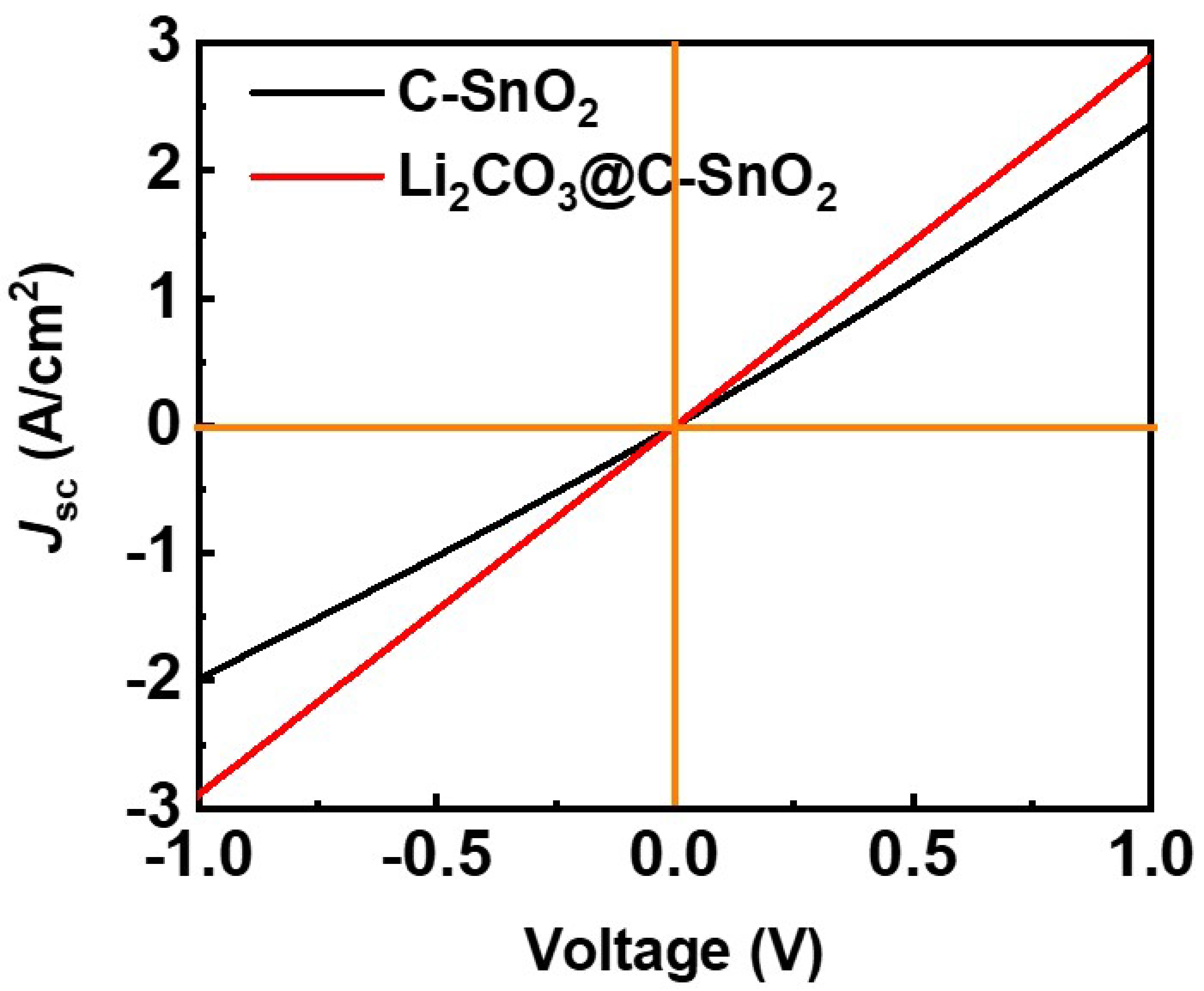


**Fig. S3** *J-V* characteristics of different ETL (The sample structure is FTO/ETL/Ag)


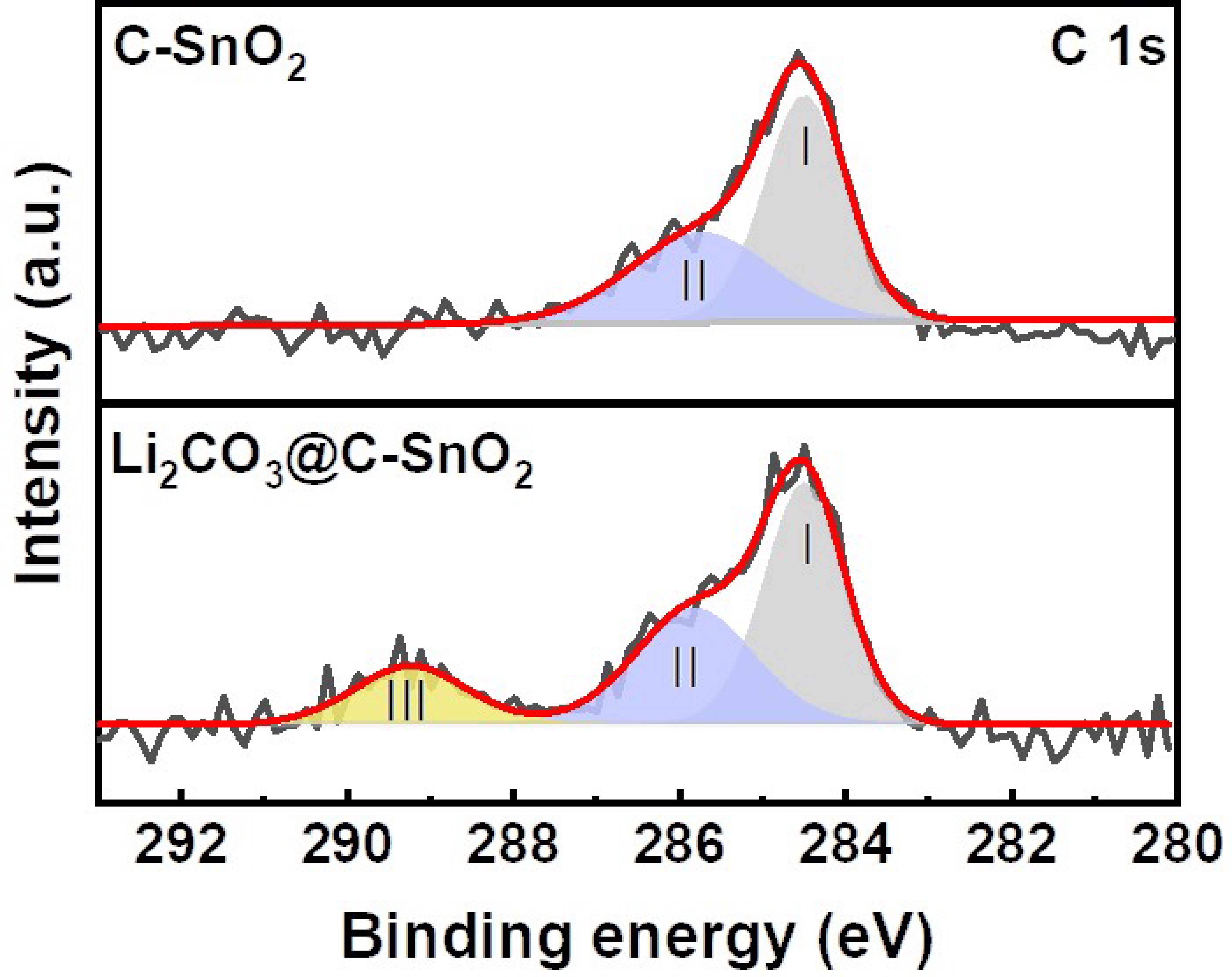


**Fig. S4** C 1s XPS spectra of the C-SnO_2_ and Li_2_CO_3_@C-SnO_2_


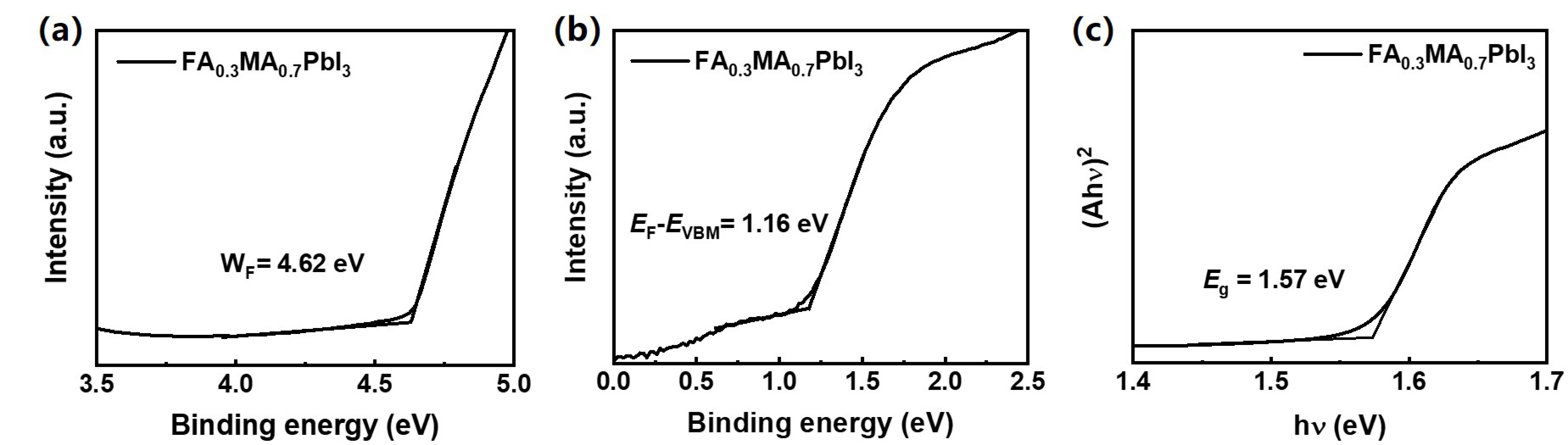


**Fig. S5** UPS spectra of perovskite film **(a)** Fermi level, **(b)** The energy gaps between the Fermi level with the valence band (*E*_F_-*E*_VBM_). **(c)** Tauc plot of the UV-vis spectra of perovskite film


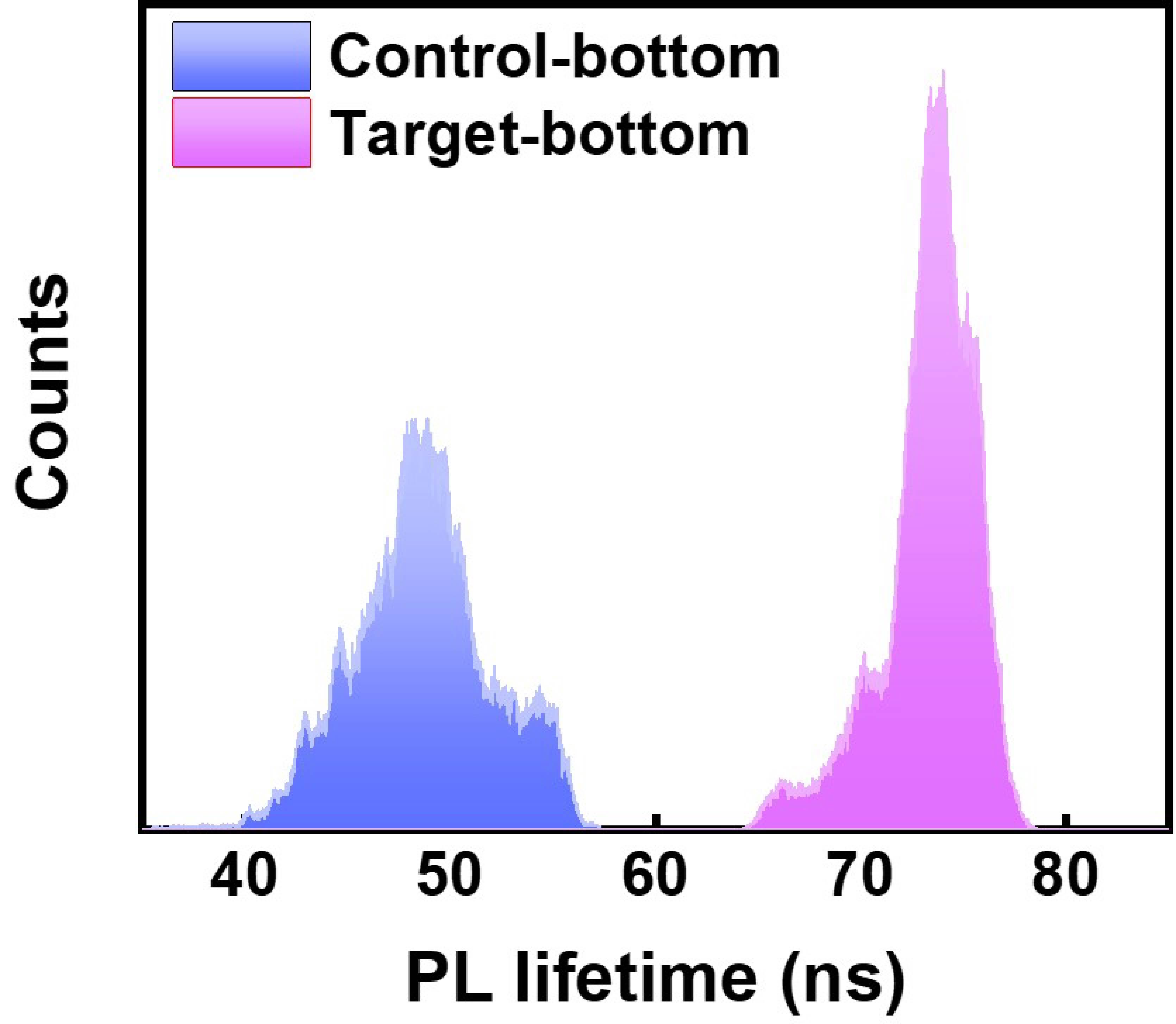


**Fig. S6** PL lifetime histogram of the bottom surface of the perovskite films


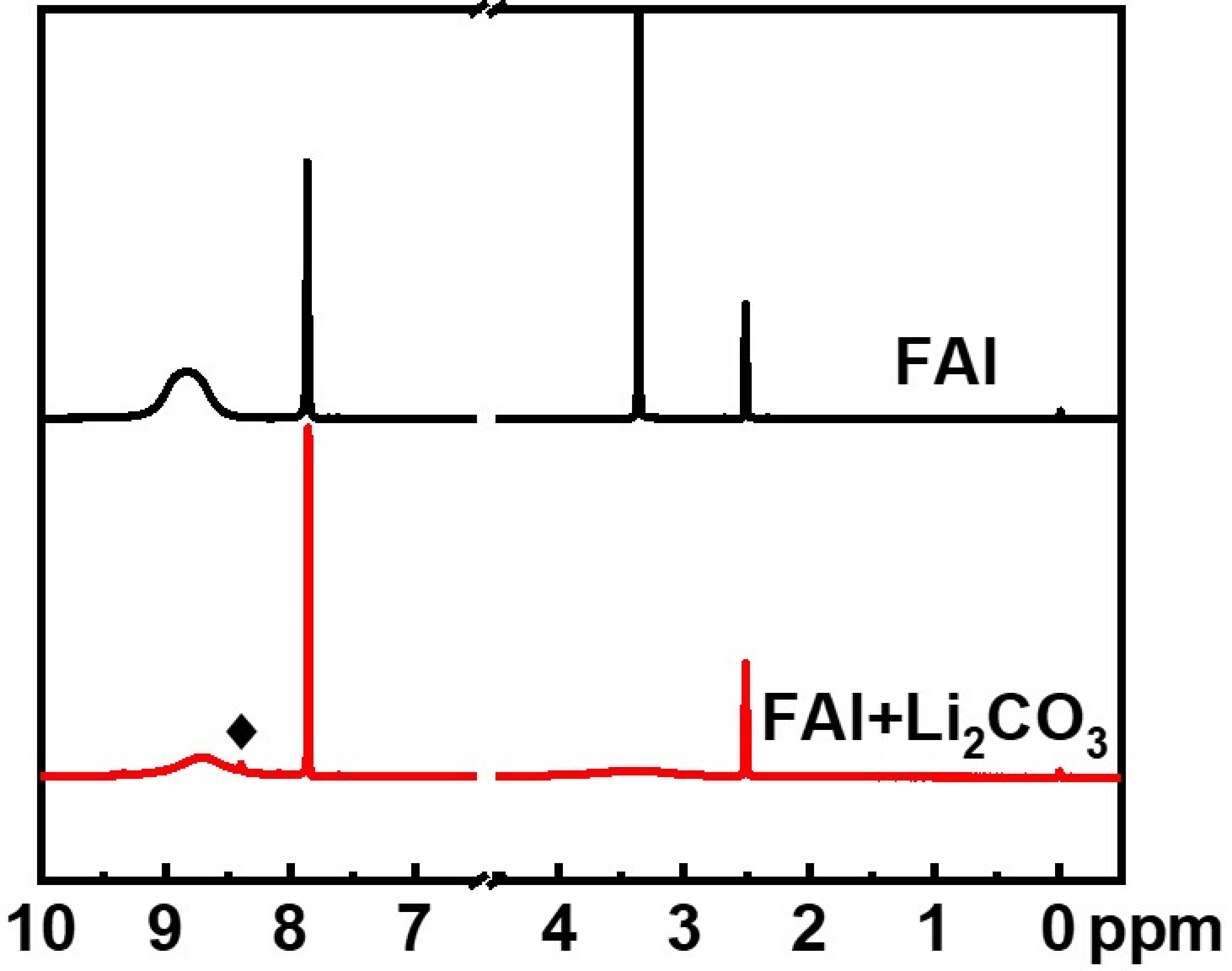


**Fig. S7** ^1^H NMR of FAI and FAI+Li_2_CO_3_


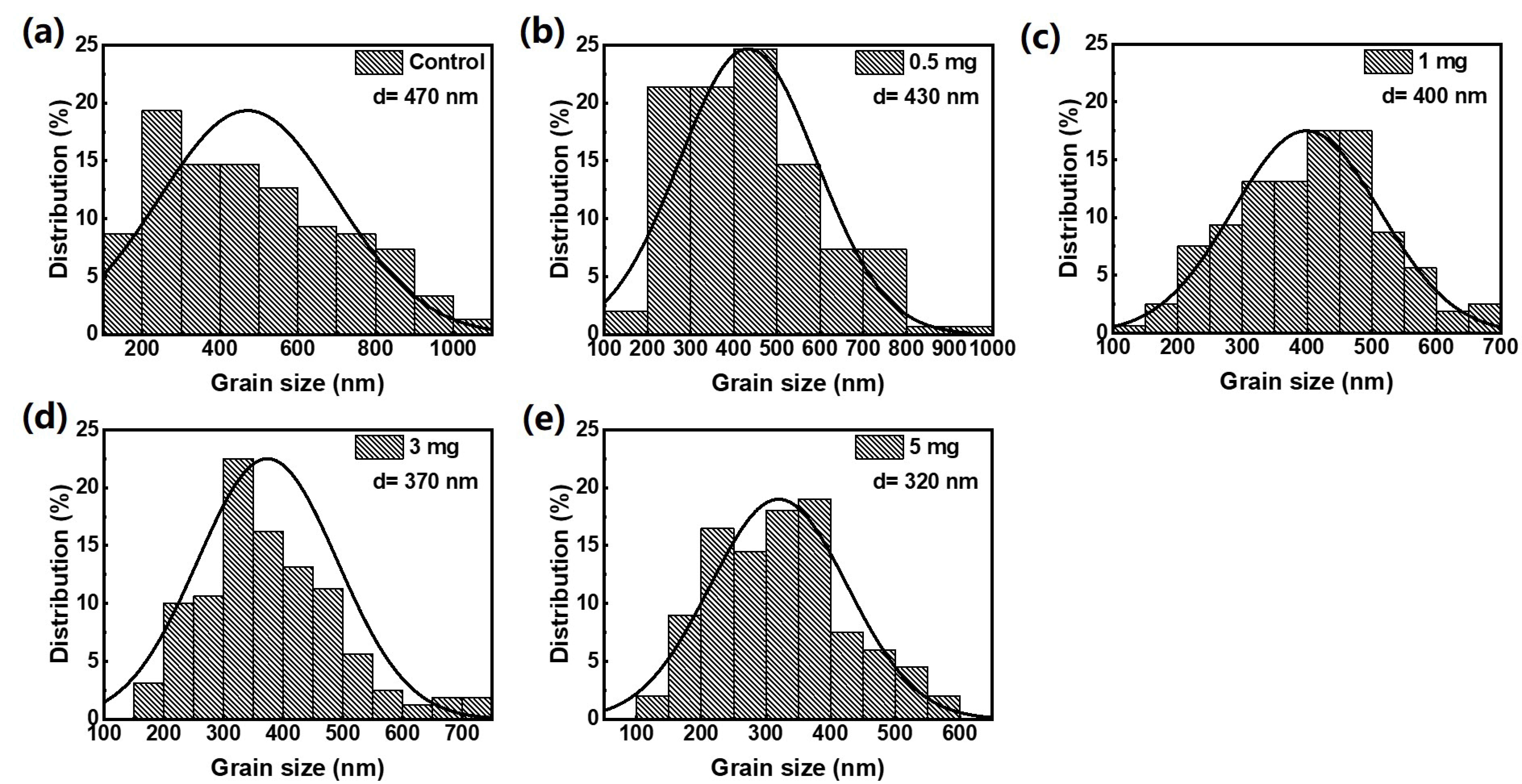


**Fig. S8** The statistical histograms of grain size distribution of perovskites on C-SnO_2_ substrates with various Li_2_CO_3_ modification conditions


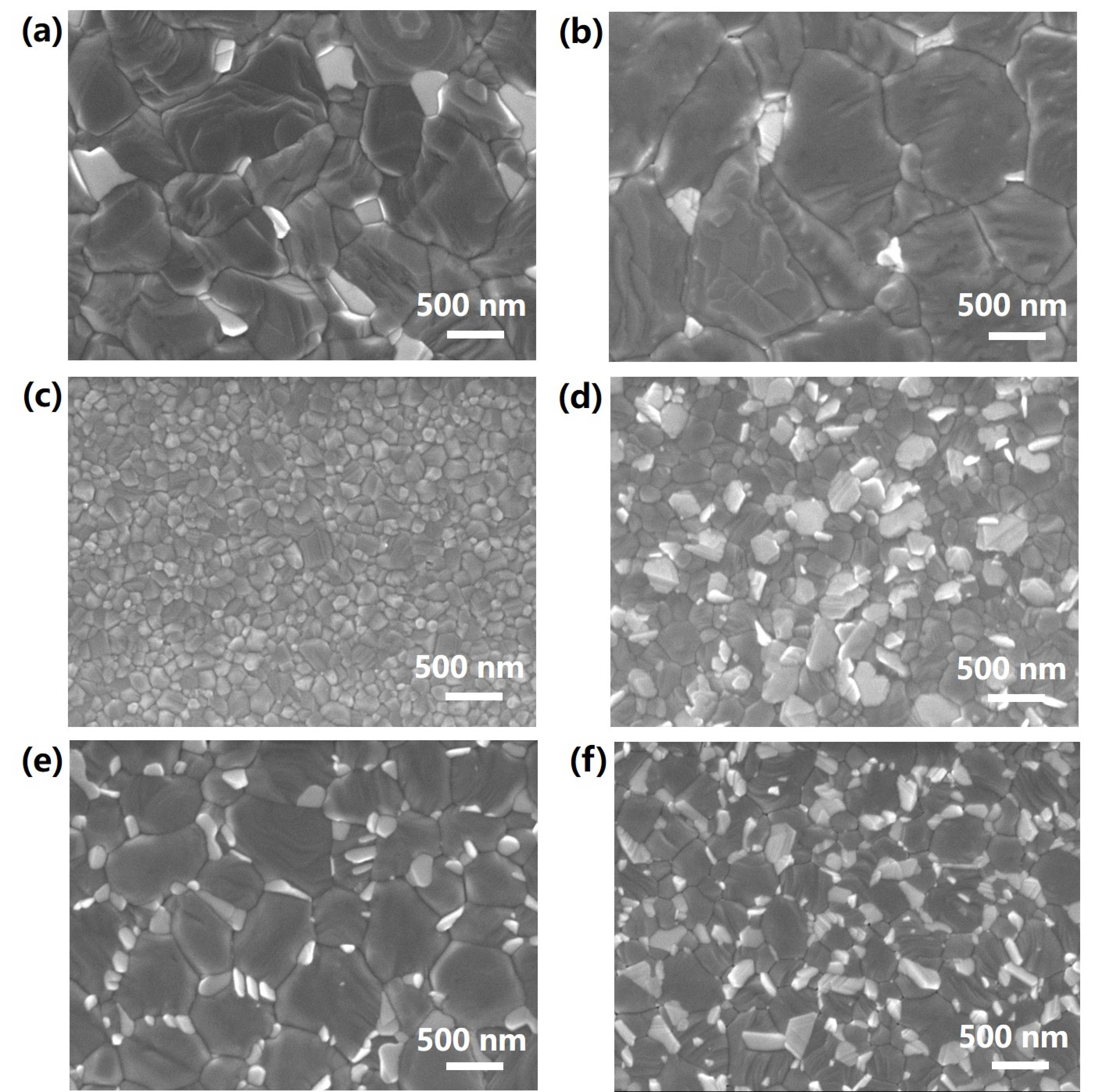


**Fig. S9** Top view SEM images of **(a)** C-SnO_2_/FAPbI_3_, **(b)** Li_2_CO_3_@C-SnO_2_/FAPbI_3_, **(c)** C-SnO_2_/MAPbI_3_, **(d)** Li_2_CO_3_@C-SnO_2_/MAPbI_3_, **(e)** C-SnO_2_/FA_0.3_MA_0.7_PbI_3_ and **(f)** [Li_2_CO_3_@C-SnO_2_/FA_0.3_MA_0.7_PbI_3_](mailto:Li2CO3@C-SnO2/FA0.3MA0.7PbI3)


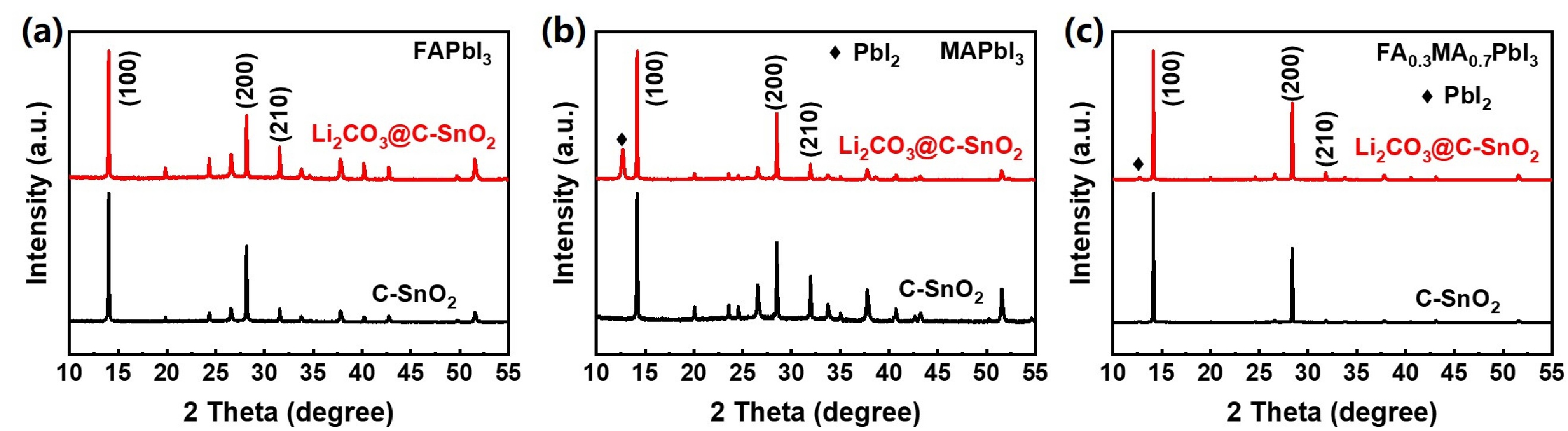


**Fig. S10** XRD patterns of perovskite films with various compositions prepared on pristine and Li_2_CO_3_ modified SnO_2_ ETLs **(a)** FAPbI_3_, **(b)** MAPbI_3_, **(c)** FA_0.3_MA_0.7_PbI_3_


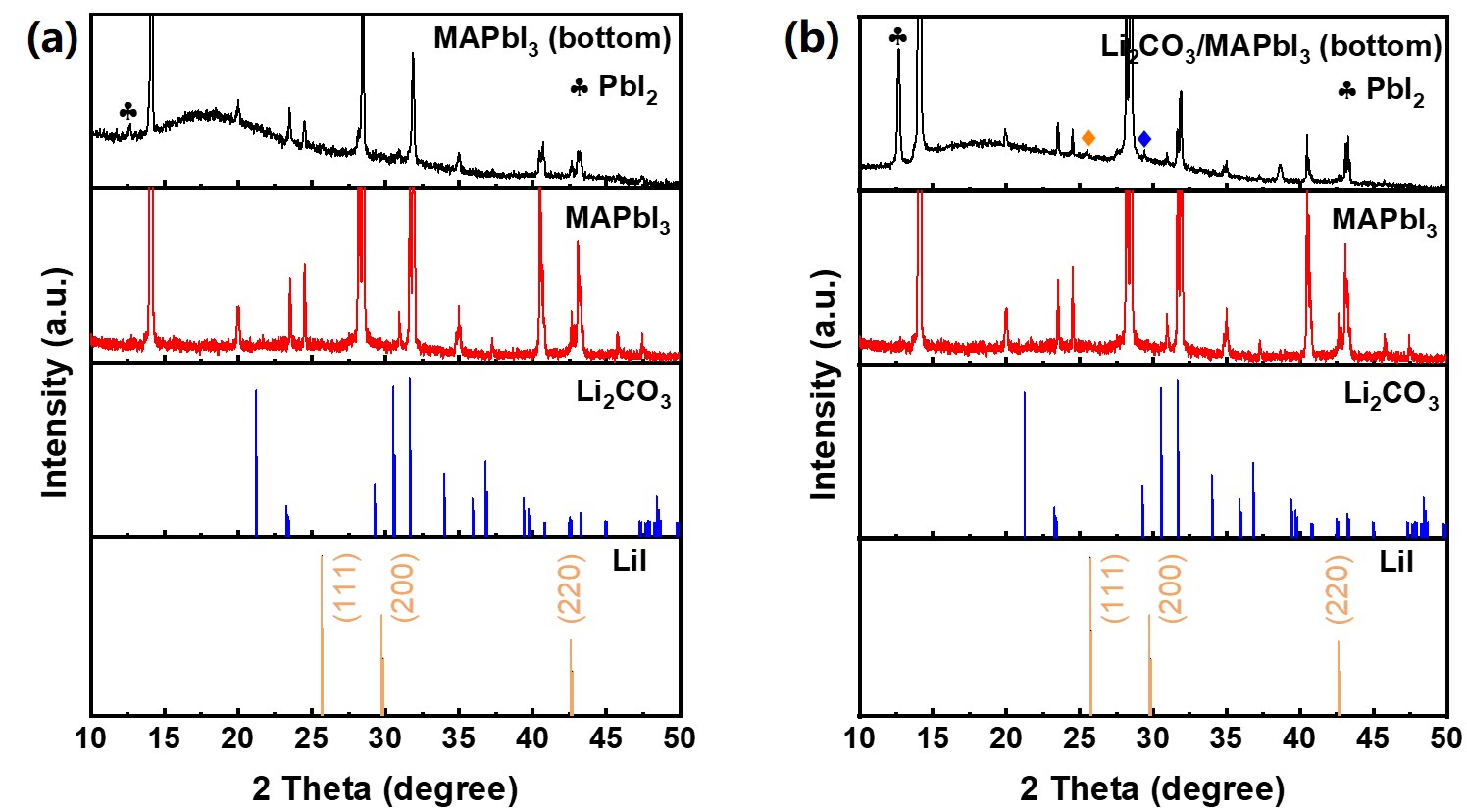


**Fig. S11** XRD patterns of the bottom interfaces of MAPbI_3_ films prepared on pristine and Li_2_CO_3_ modified substrates


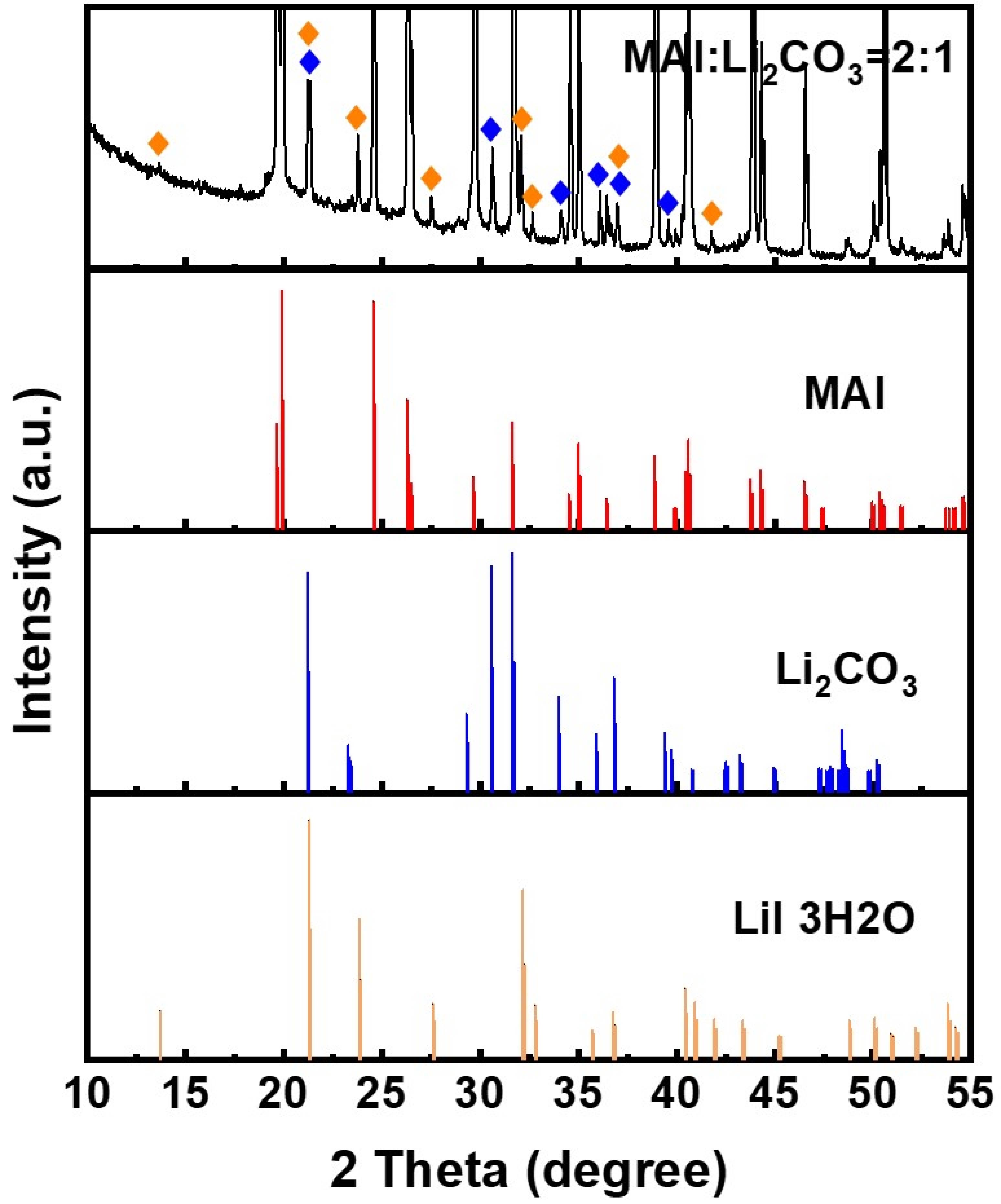


**Fig. S12** Powder XRD pattern of the solid mixture of MAI and Li_2_CO_3_ at a molar ratio of 2:1 after subjected to thorough grinding for 10 minutes and annealing at 100°C for 20 minutes


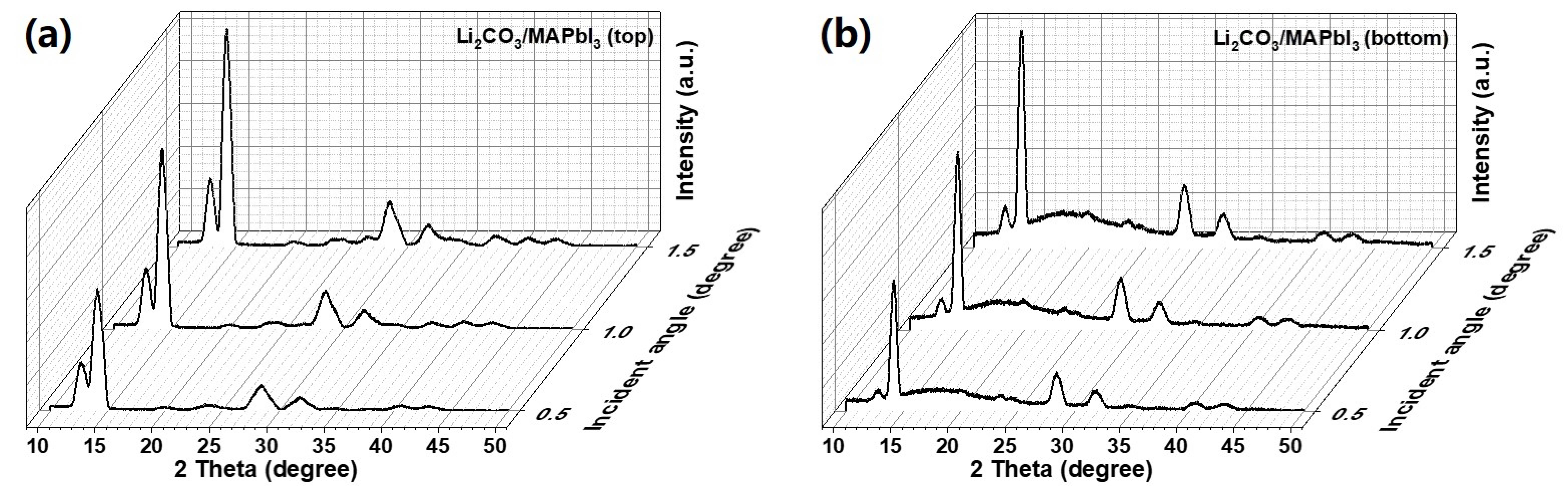


**Fig. S13** GIXRD patterns of the top surface and bottom interface of MAPbI_3_ films prepared on Li_2_CO_3_ modified substrates


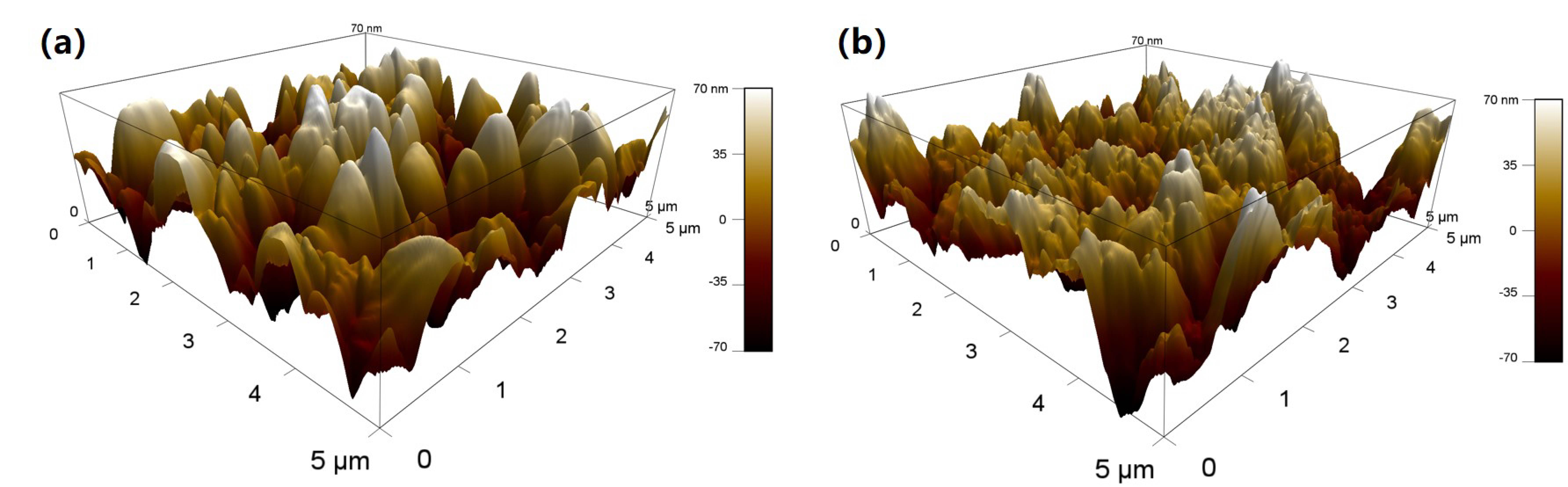


**Fig. S****14** AFM images of the perovskite films deposited on **(a)** C-SnO_2_ and **(b)** Li_2_CO_3_@C-SnO_2_


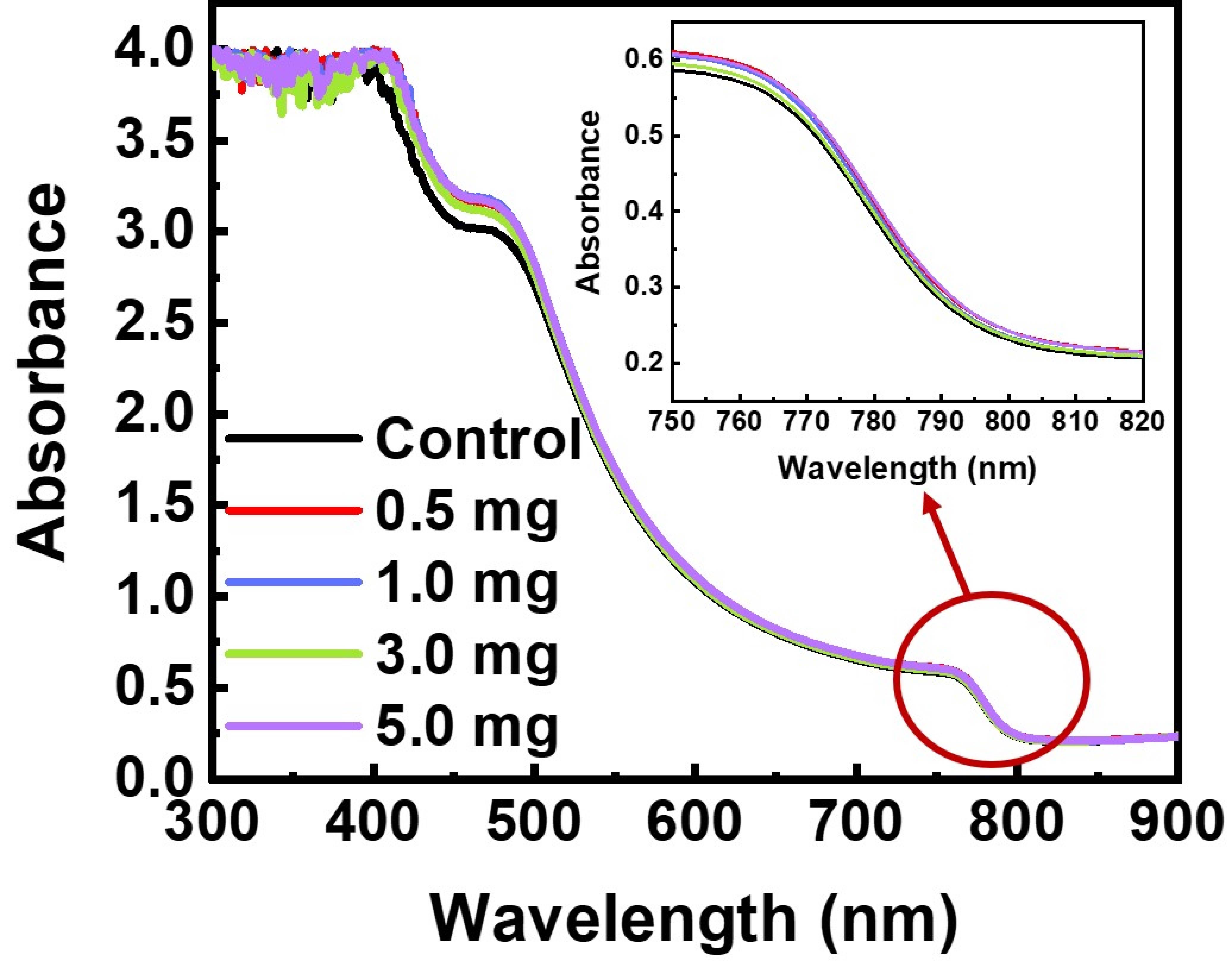


**Fig. S15** UV-Vis absorption spectra of perovskite films deposited on C-SnO_2_ modified by different concentrations of Li_2_CO_3_


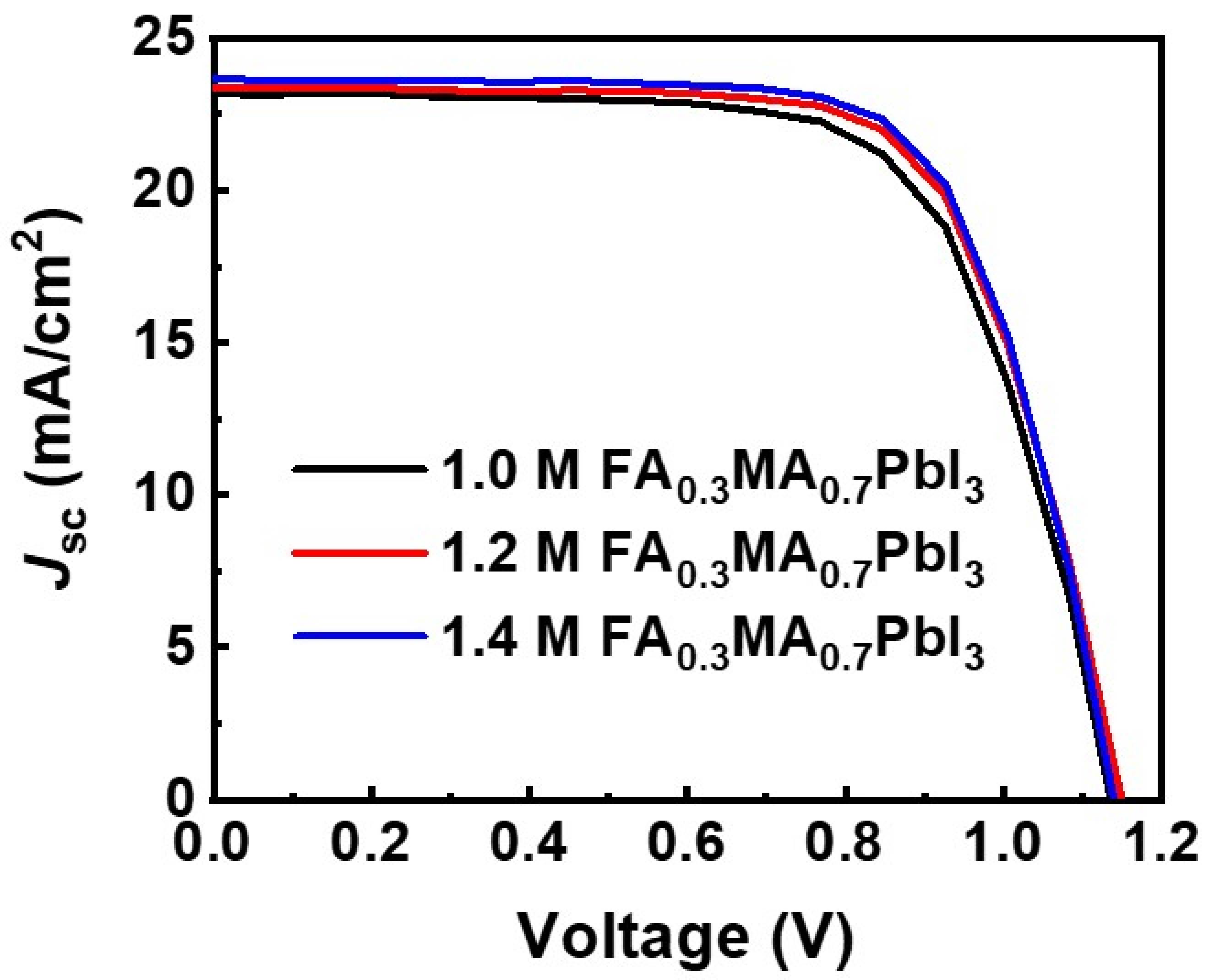


**Fig. S16** The *J−V* curves of the devices with different perovskite film thickness based on Li_2_CO_3_ modified C-SnO_2_


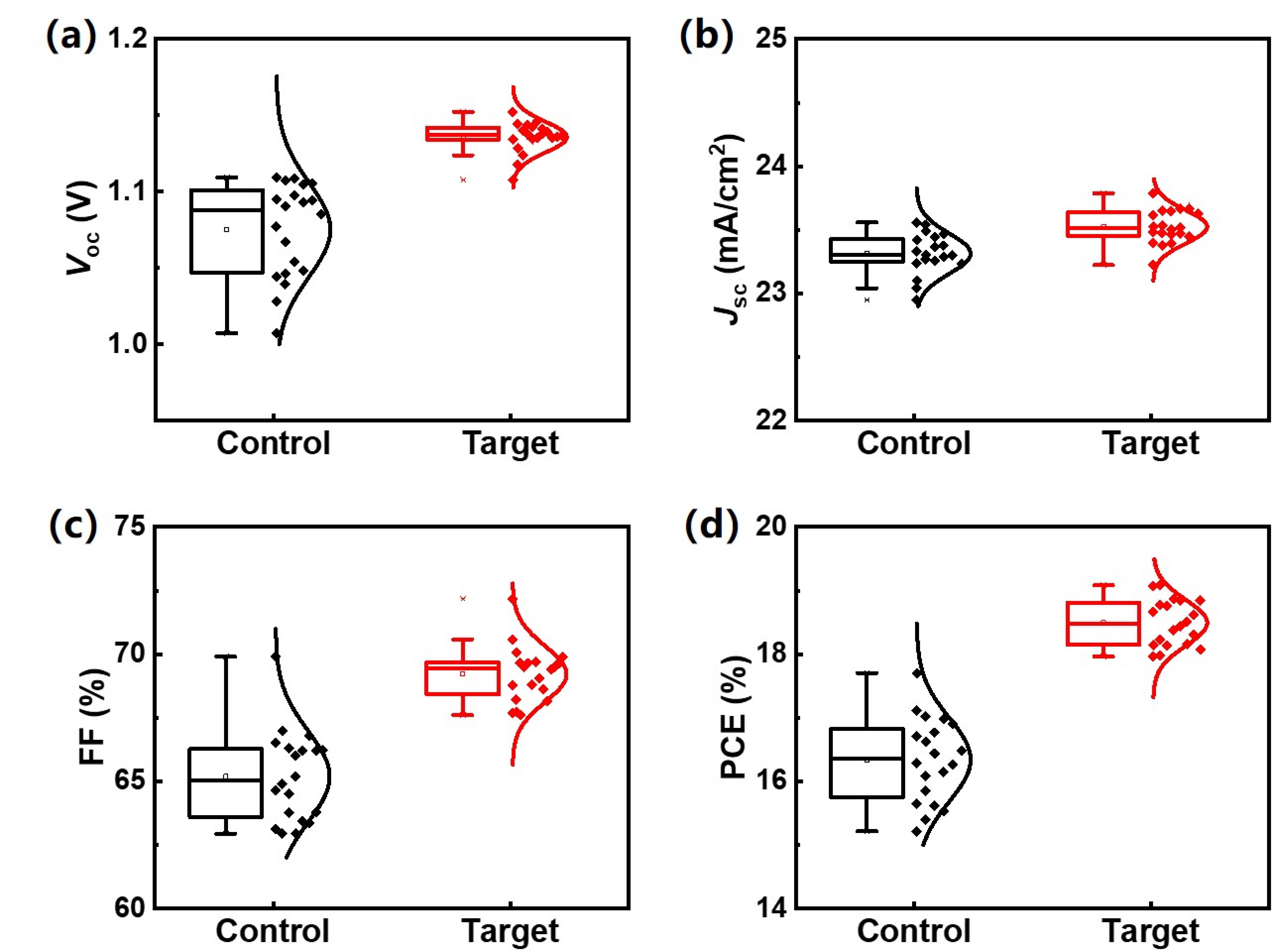


**Fig. 17** Device performance characterization. The statistic photovoltaic parameters of: **(a)** *V*_oc_, **(b)** *J*_sc_, **(c)** FF, and **(d)** PCE of the control and target devices


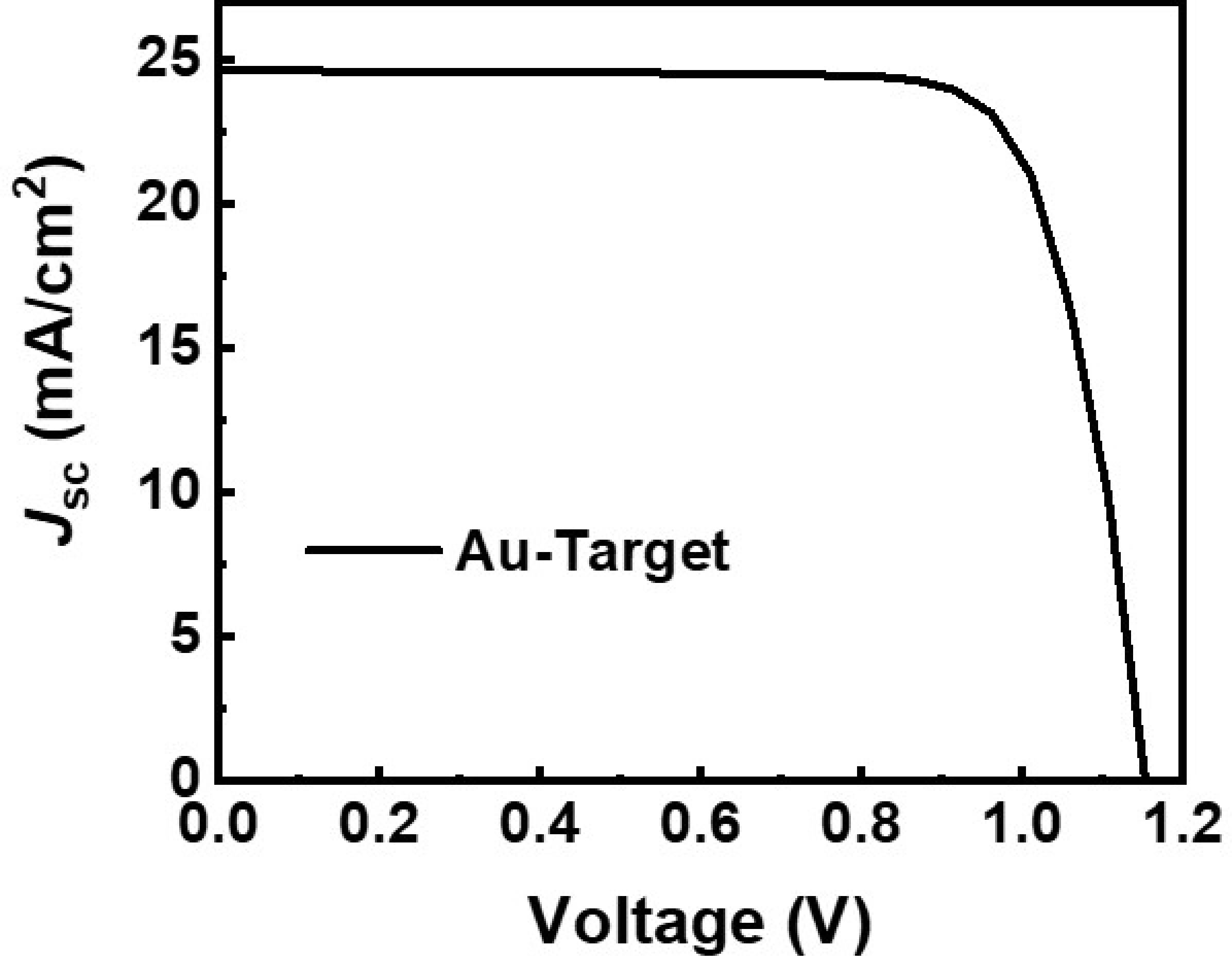


**Fig. S18** The *J−V* curve of device with the structure of Li_2_CO_3_@C-SnO_2_/perovskite/Spiro-OMeTAD/Au

**
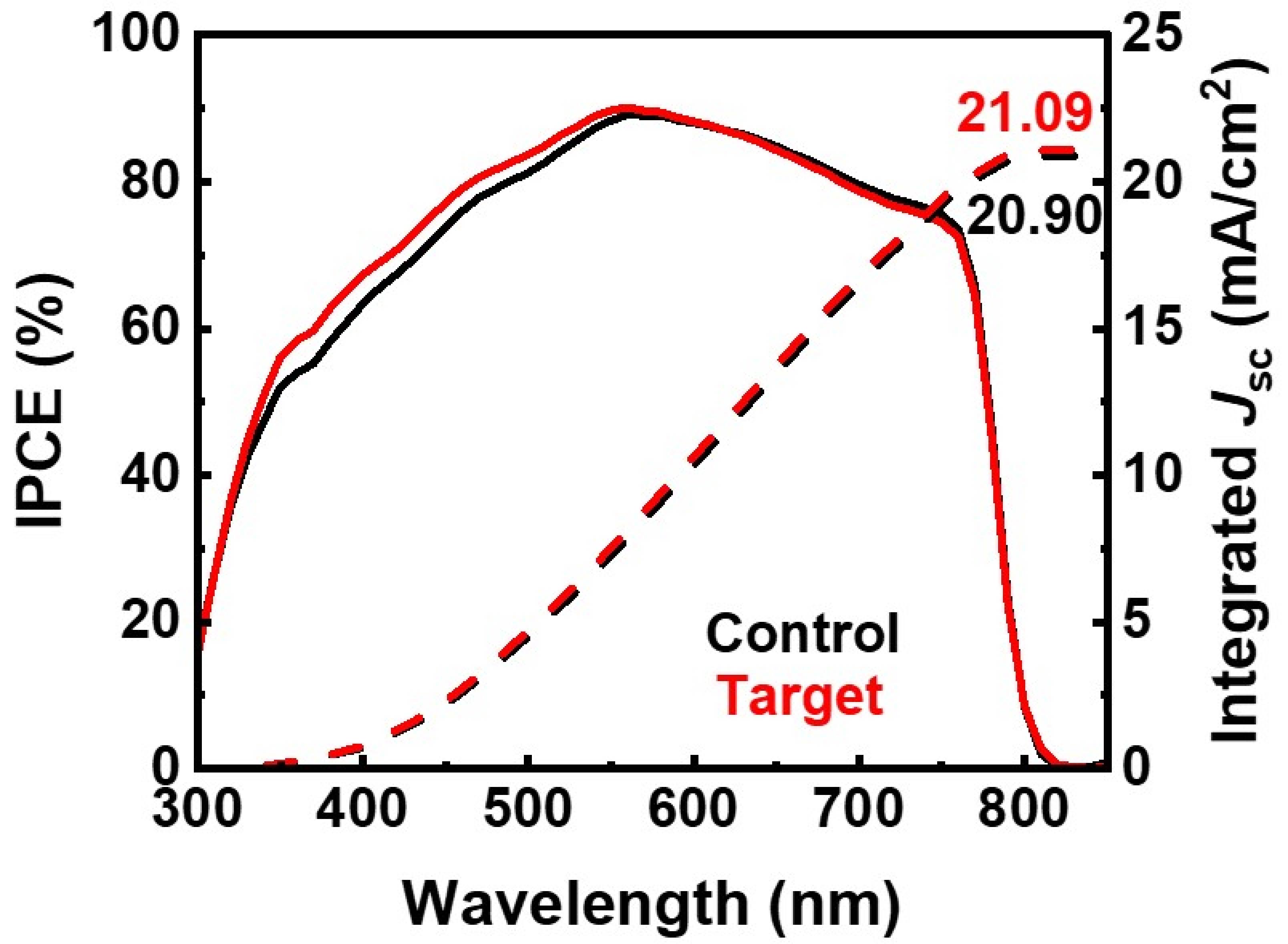
**

**Fig. S19** IPCE spectra of control and target PSCs


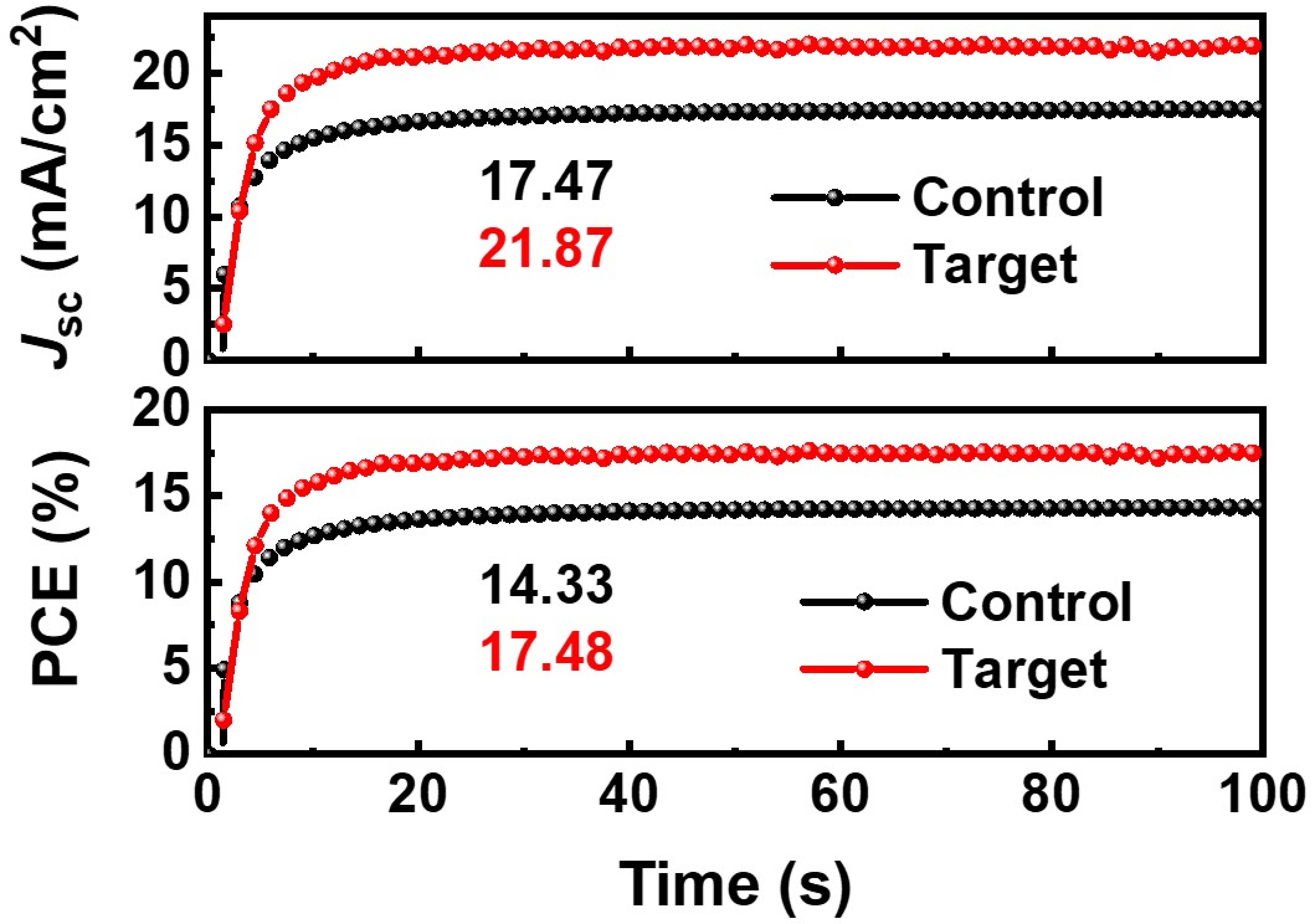


**Fig. S20** Steady output of the control device and target device


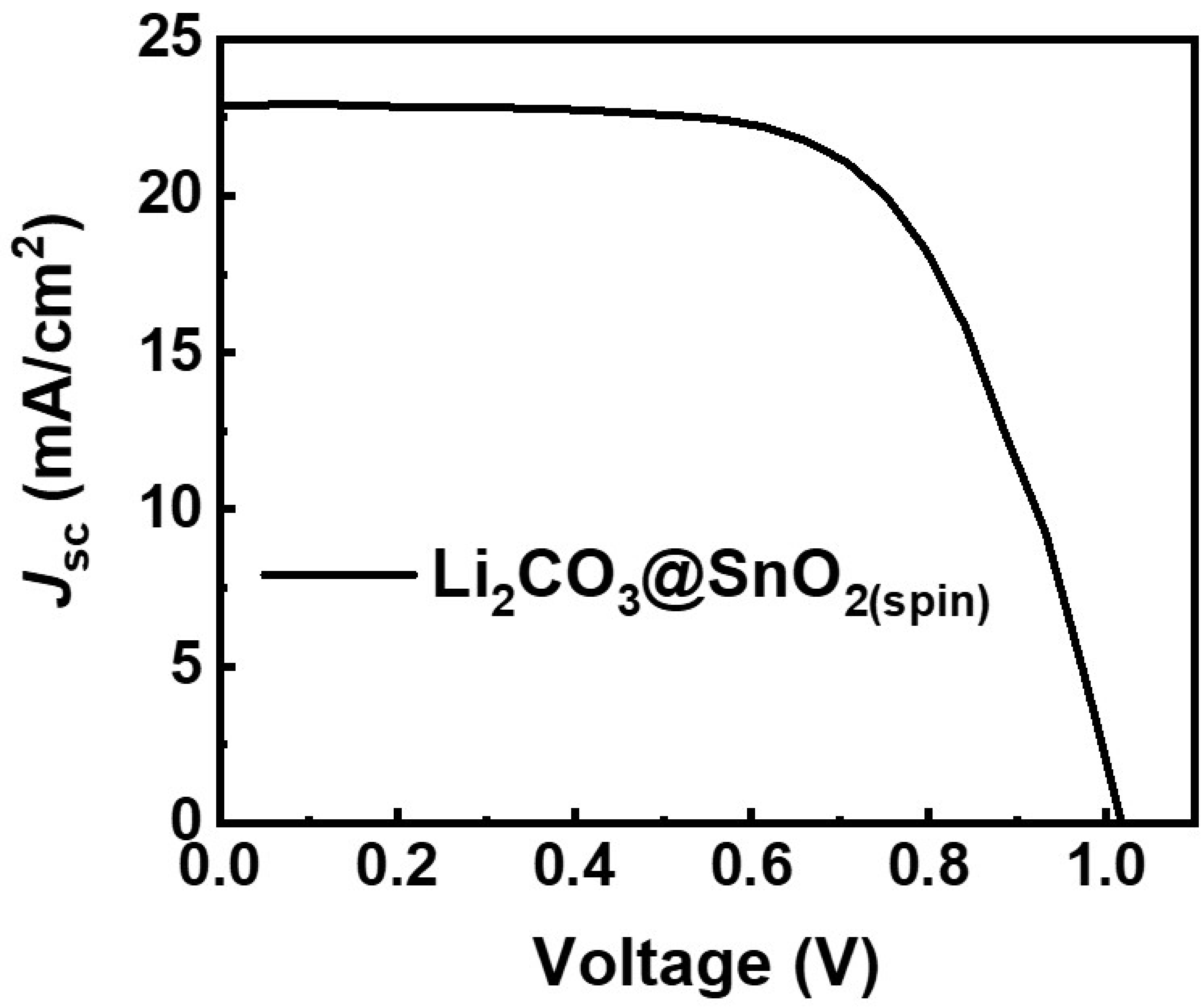


**Fig. S21** The *J−V* curve of the PSC based on Li_2_CO_3_@SnO_2(spin)_


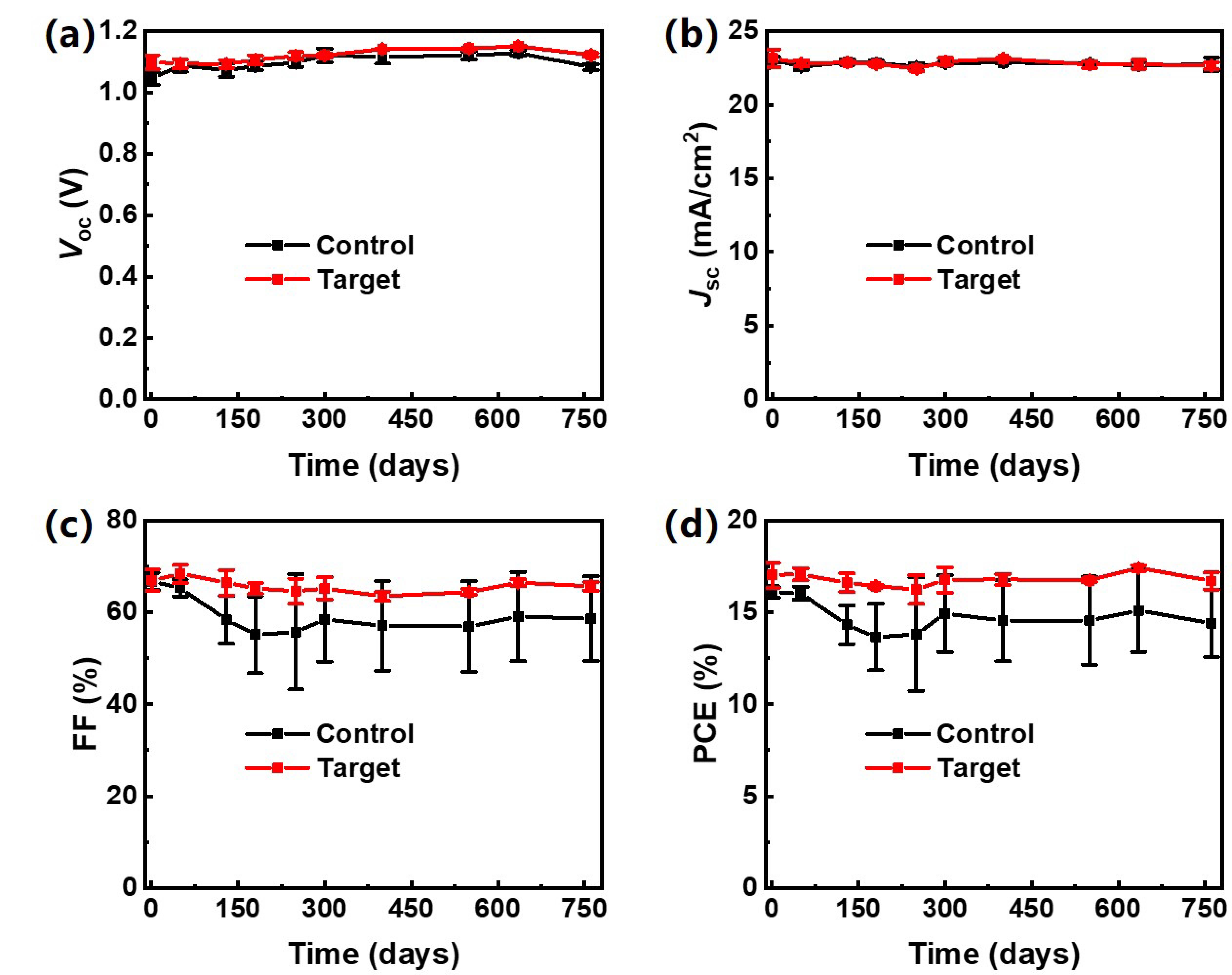


**Fig. S22** Long-term stability under ambient condition (temperature~15-30°C, relative humidity~20%) without any encapsulation **(a)** *V*_oc_, **(b)** *J*_sc_, **(c)** FF and **(d)** PCE


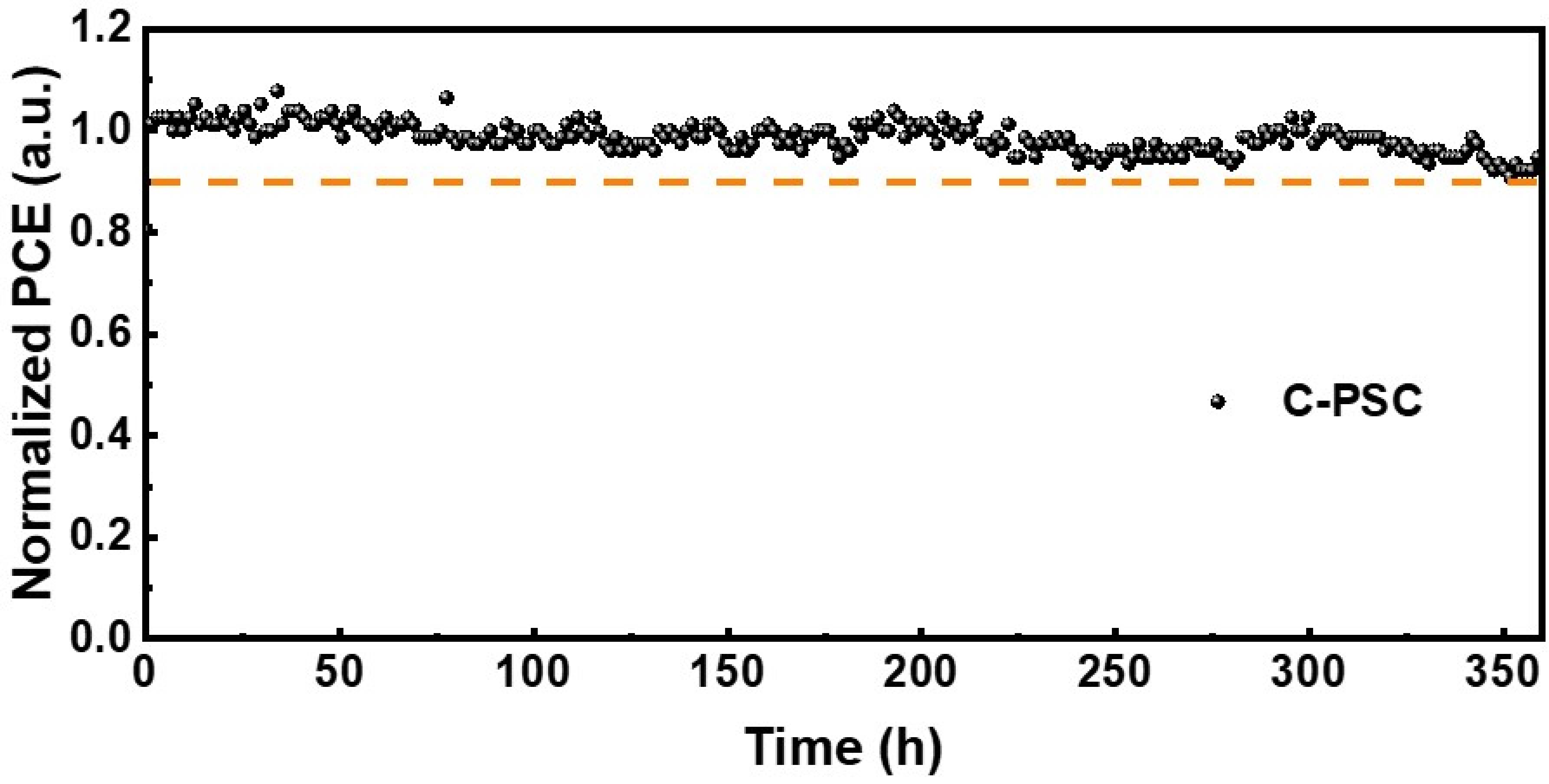


**Fig. S23** Normalized PCE of C-PSC device tracked at maximum power point (MPP) under continuous 1 sun illumination


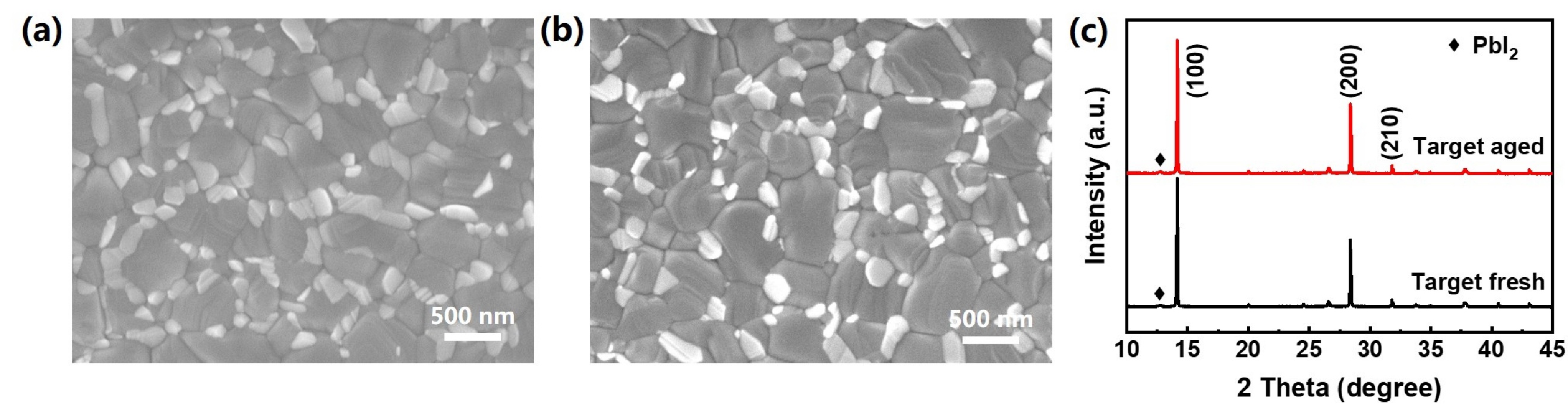


**Fig. S24** SEM images of target sample **(a)** fresh, **(b)** after aging for 30 days. **(c)** XRD patterns of the fresh and aged target sample


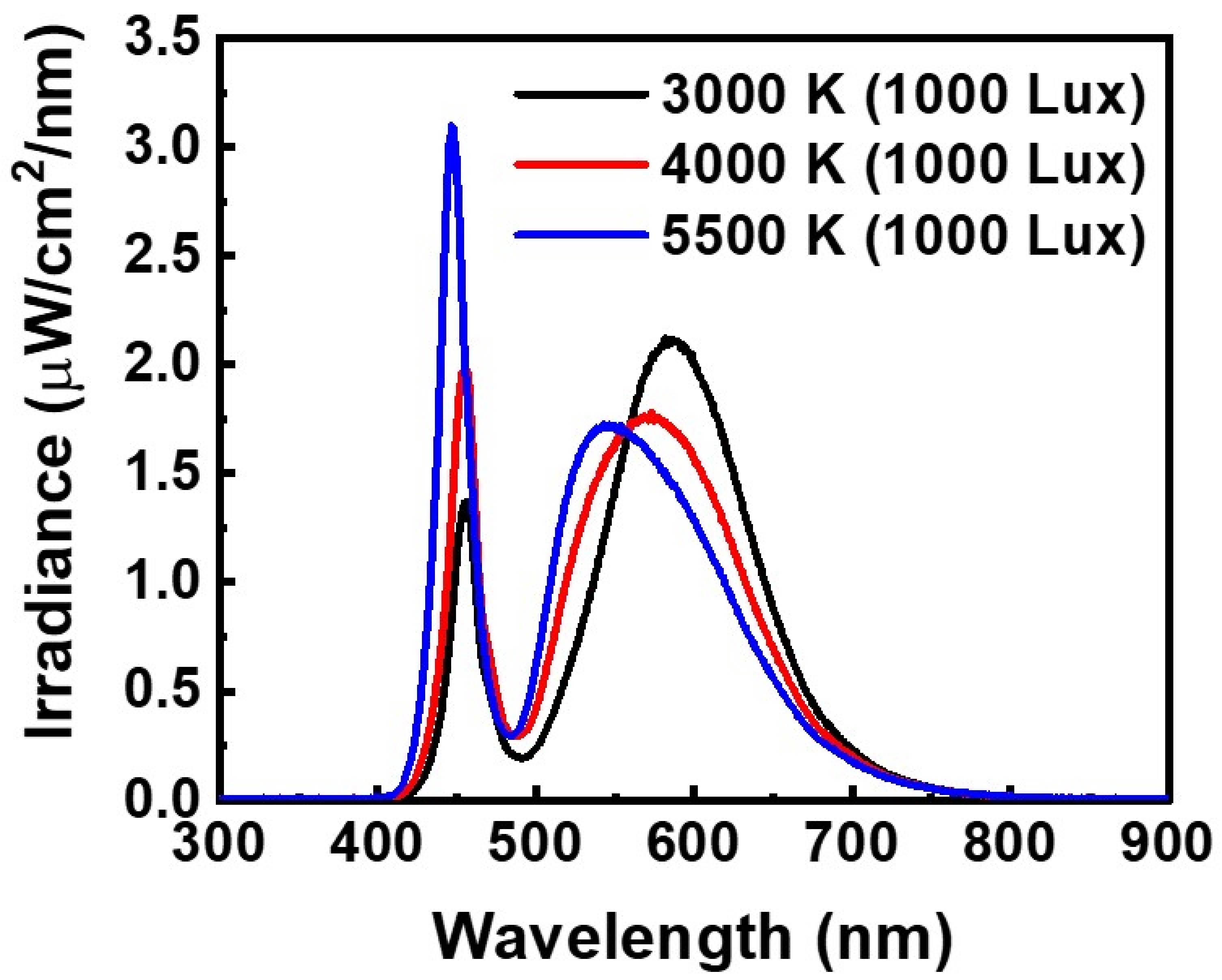


**Fig. S****25** Emission spectra of the indoor light source at different color temperatures


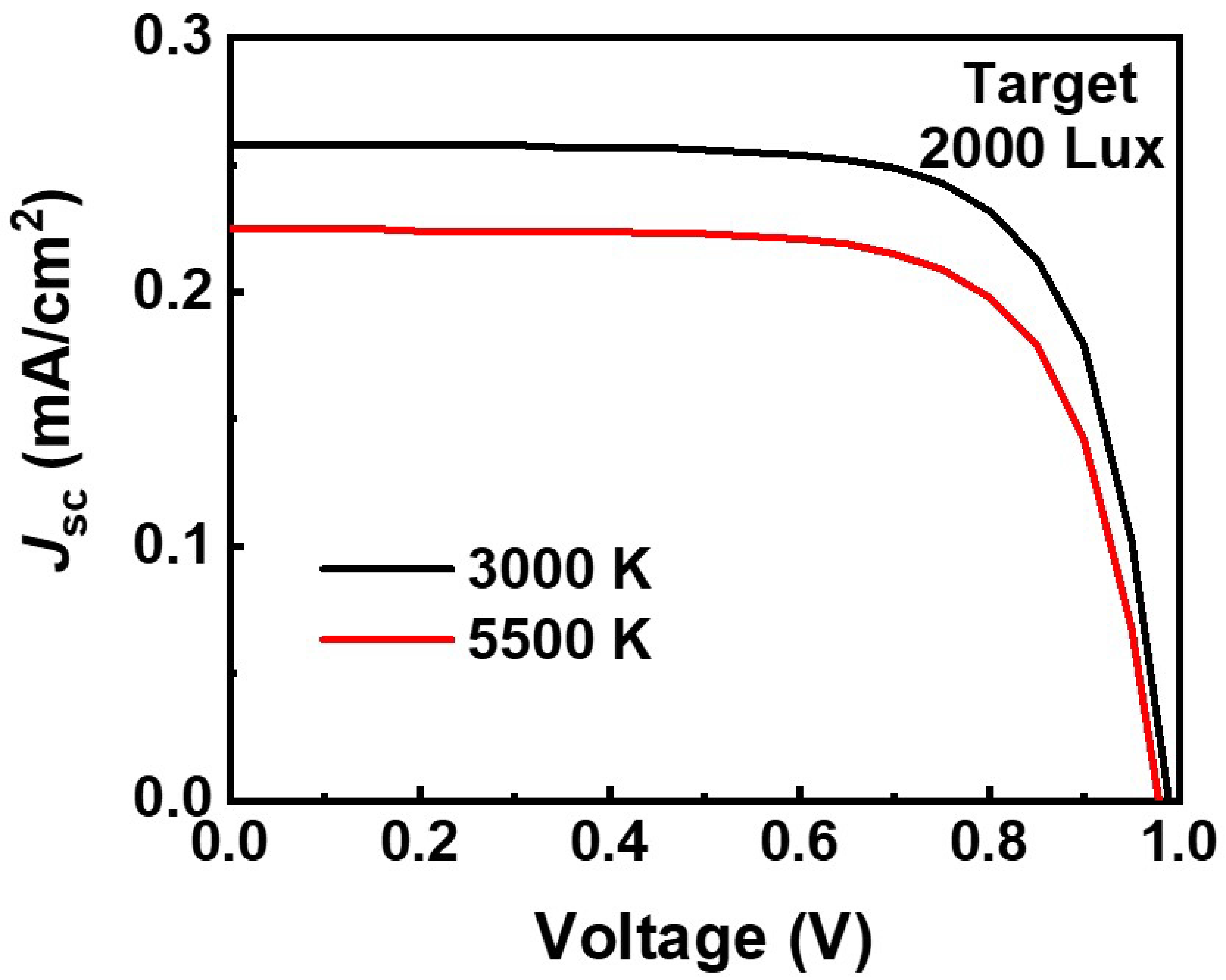


**Fig. S26** The *J−V* curves of target devices measured under LED illumination at 2000 lux with the color temperature of 3000 K and 5500 K, respectively


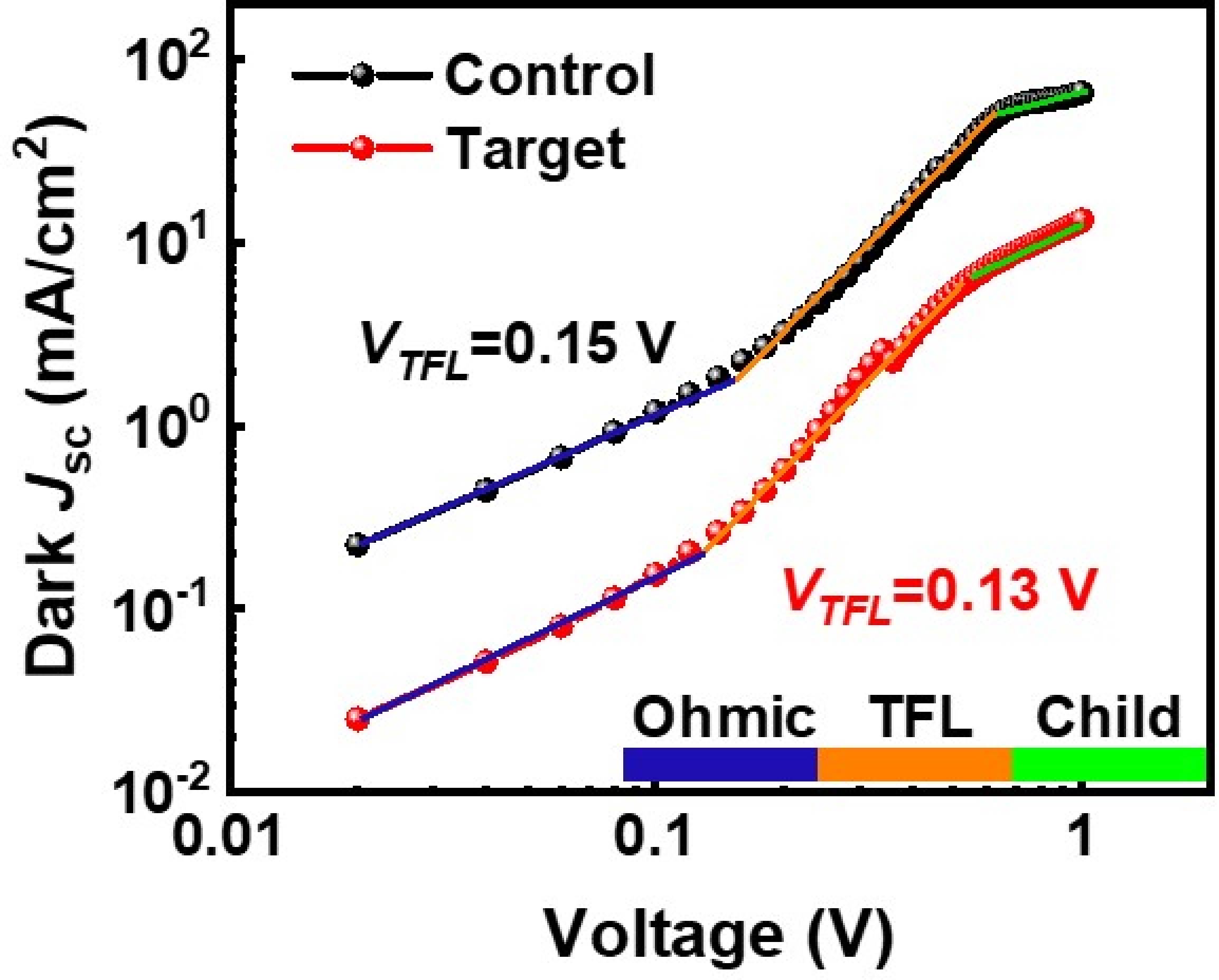


**Fig. S2****7** SCLC plots of the electron-only devices


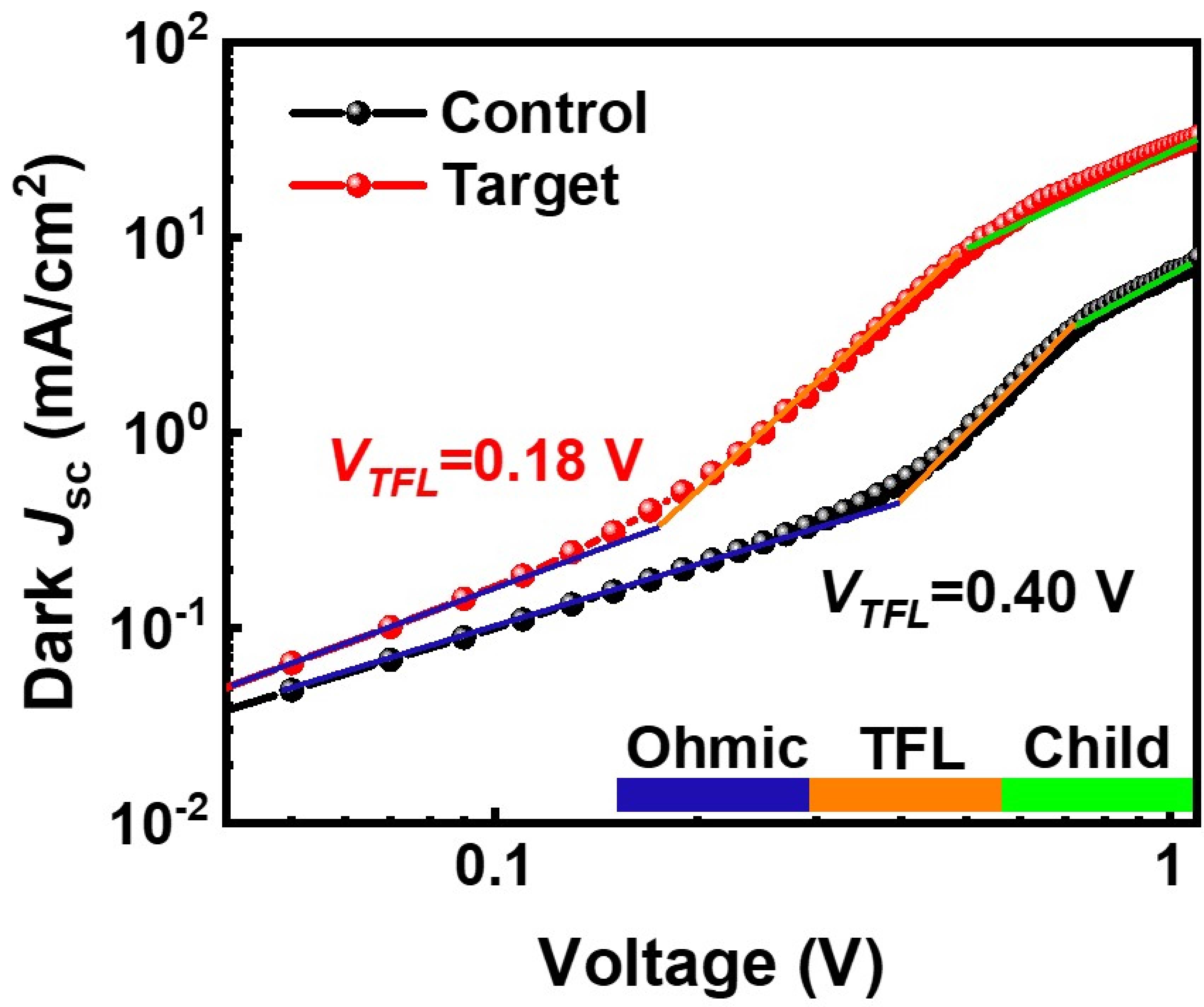


**Fig. S28** SCLC plots of the hole-only devices


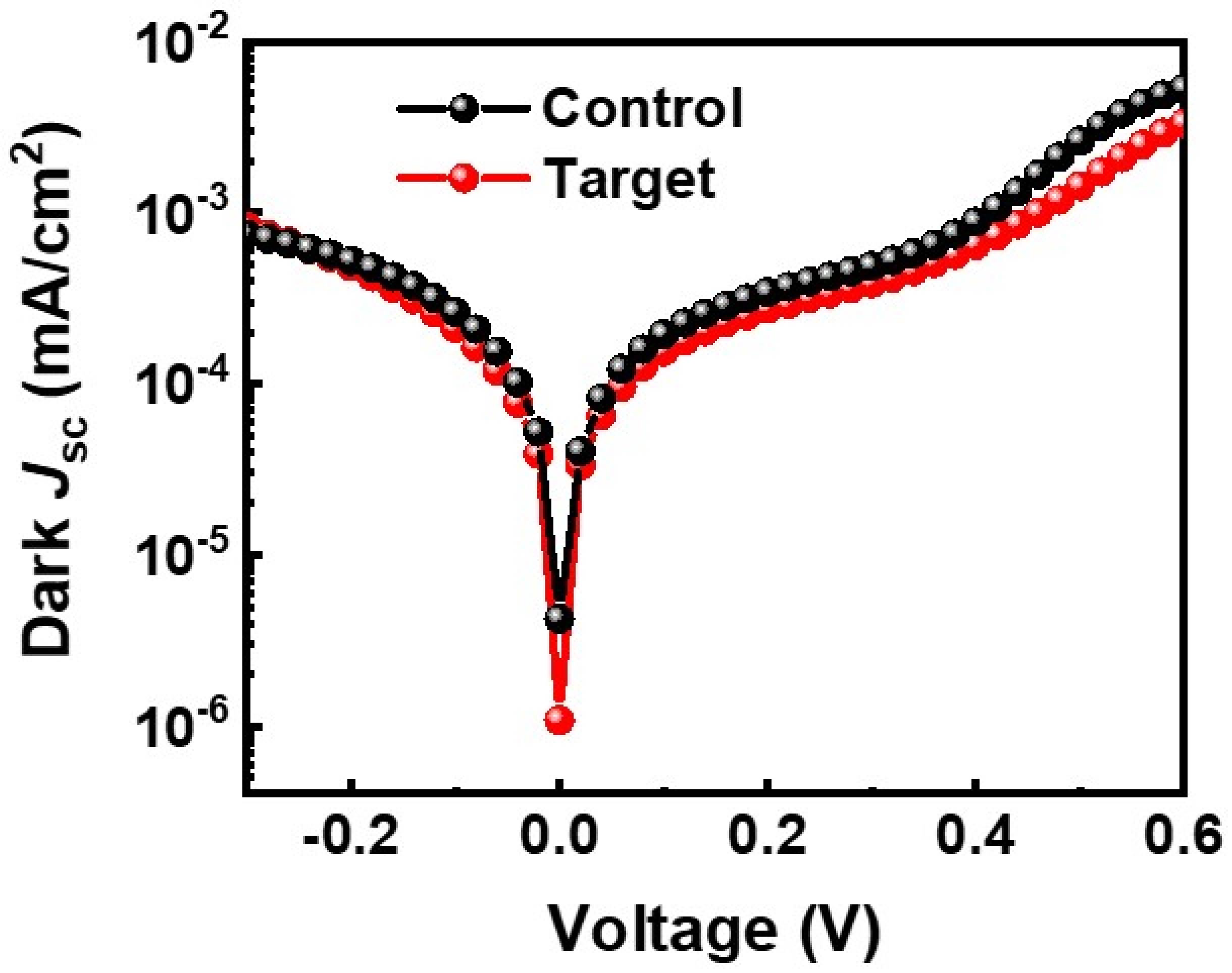


**Fig. S29** *J−V* curves of the control and target devices under dark condition


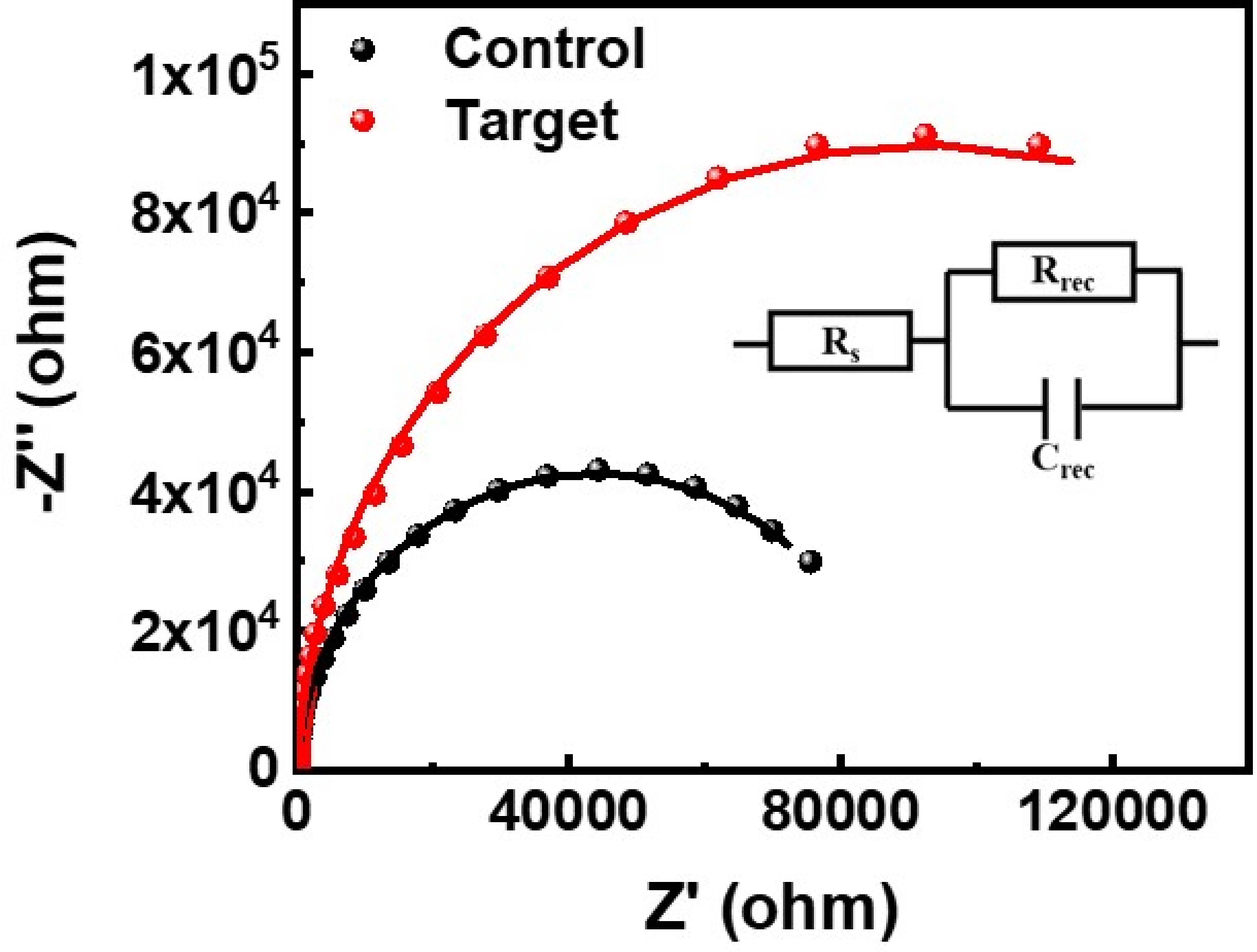


**Fig. S30** Nyquist plots of control and target devices measured in the dark with an applied bias voltage *V*_bias_ = 0.4 V

**Table S1** The fitted peak area of PbI_2_ (12.7°) and MAPbI_3_ (14.1°) signature diffraction peaks of the MAPbI_3_ films prepared on Li_2_CO_3_ modified substrates obtained using GIXRD. The GIXRD patterns were collected from the top surface and the bottom interface of the perovskite films

| **Sample** | **Incident angle (degree)** | **PbI_2_ peak area (a.u.)** | **Perovskite peak area (a.u.)** | **PbI_2_ peak area /Perovskite peak area** |
| --- | --- | --- | --- | --- |
| Li_2_CO_3_/MAPbI_3_  top surface | 0.5 | 30524 | 125432 | 0.243 |
|  | 1.0 | 42289 | 177995 | 0.238 |
|  | 1.5 | 53665 | 212919 | 0.252 |

| **Sample** | **Incident angle (degree)** | **PbI_2_ peak area (a.u.)** | **Perovskite peak area (a.u.)** | **PbI_2_ peak area /Perovskite peak area** |
| --- | --- | --- | --- | --- |
| Li_2_CO_3_/MAPbI_3_  bottom interface | 0.5 | 4654 | 72691 | 0.064 |
|  | 1.0 | 9480 | 103280 | 0.091 |
|  | 1.5 | 15196 | 128111 | 0.119 |

**Table S2** The statistics of photovoltaic parameters of devices based on the C-SnO_2_ with different Li_2_CO_3_ modification conditions

| **Concentration**  **(mg/mL)** | **Sample No.** | ***J*_sc_ (mA/cm^2^)** | ***V*_oc_ (V)** | **FF (%)** | **PCE (%)** |
| --- | --- | --- | --- | --- | --- |
| Control | 1 | 22.78 | 1.037 | 61.6 | 14.5 |
|  | 2 | 22.79 | 0.997 | 64.1 | 14.6 |
|  | 3 | 23.45 | 1.028 | 60.3 | 14.5 |
|  | 4 | 23.23 | 1.036 | 64.2 | 15.4 |
|  | 5 | 22.26 | 0.972 | 61.7 | 13.3 |
|  | 6 | 22.93 | 0.983 | 60.7 | 13.7 |
|  | 7 | 23.14 | 0.985 | 59.1 | 13.5 |
|  | 8 | 22.91 | 1.001 | 65.8 | 15.1 |
|  | 9 | 22.94 | 0.993 | 63.6 | 14.5 |
|  | 10 | 23.10 | 0.997 | 61.7 | 14.2 |
|  | 11 | 22.77 | 1.003 | 61.8 | 14.1 |
|  | average | 22.94 | 1.003 | 63.2 | 14.3 |
| 0.5 | 1 | 22.64 | 1.054 | 63.1 | 15.1 |
|  | 2 | 22.71 | 1.059 | 61.9 | 14.9 |
|  | 3 | 22.33 | 1.043 | 64.6 | 15.0 |
|  | 4 | 23.30 | 1.058 | 68.2 | 16.8 |
|  | 5 | 22.84 | 1.057 | 64.9 | 15.7 |
|  | 6 | 23.40 | 1.065 | 64.0 | 15.9 |
|  | 7 | 23.08 | 1.052 | 62.3 | 15.1 |
|  | 8 | 23.10 | 1.053 | 61.3 | 14.9 |
|  | 9 | 22.99 | 1.003 | 63.9 | 14.7 |
|  | 10 | 23.02 | 1.003 | 63.2 | 14.6 |
|  | 11 | 23.00 | 1.024 | 64.4 | 15.7 |
|  | average | 22.95 | 1.043 | 63.8 | 15.3 |
| 1.0 | 1 | 22.34 | 0.975 | 57.2 | 12.5 |
|  | 2 | 22.42 | 1.059 | 62.4 | 14.8 |
|  | 3 | 22.83 | 1.069 | 60.6 | 14.7 |
|  | 4 | 22.83 | 1.068 | 59.6 | 14.5 |
|  | 5 | 22.76 | 1.077 | 59.7 | 14.7 |
|  | 6 | 23.03 | 1.043 | 63.6 | 15.3 |
|  | 7 | 23.12 | 1.048 | 64.8 | 15.7 |
|  | 8 | 22.96 | 1.062 | 61.4 | 15.0 |
|  | 9 | 22.43 | 1.065 | 61.0 | 14.6 |
|  | 10 | 22.42 | 0.971 | 60.0 | 13.1 |
|  | 11 | 22.93 | 1.025 | 62.3 | 14.6 |
|  | average | 22.73 | 1.042 | 61.1 | 14.5 |
| 3.0 | 1 | 23.01 | 1.076 | 61.3 | 15.2 |
|  | 2 | 23.07 | 1.080 | 63.5 | 15.8 |
|  | 3 | 22.97 | 1.084 | 63.2 | 15.7 |
|  | 4 | 22.95 | 1.088 | 63.6 | 15.9 |
|  | 5 | 22.40 | 1.032 | 61.3 | 14.2 |
|  | 6 | 23.09 | 1.038 | 63.0 | 15.1 |
|  | 7 | 22.87 | 1.059 | 63.5 | 15.4 |
|  | 8 | 23.06 | 1.057 | 62.2 | 15.2 |
|  | 9 | 22.94 | 1.025 | 64.7 | 15.2 |
|  | 10 | 22.88 | 1.028 | 65.7 | 15.5 |
|  | 11 | 23.07 | 1.030 | 65.5 | 16.0 |
|  | average | 22.94 | 1.054 | 63.4 | 15.4 |
| 5.0 | 1 | 22.93 | 1.040 | 40.8 | 9.73 |
|  | 2 | 23.01 | 1.056 | 54.9 | 13.3 |
|  | 3 | 22.66 | 1.059 | 62.4 | 15.0 |
|  | 4 | 22.92 | 1.054 | 57.9 | 14.0 |
|  | 5 | 22.61 | 0.945 | 42.0 | 9.0 |
|  | 6 | 22.87 | 1.002 | 42.9 | 10.1 |
|  | 7 | 22.73 | 1.008 | 41.4 | 9.5 |
|  | 8 | 23.00 | 1.052 | 50.7 | 12.3 |
|  | 9 | 22.86 | 1.063 | 58.6 | 14.8 |
|  | 10 | 23.05 | 1.073 | 61.5 | 15.2 |
|  | 11 | 22.63 | 1.036 | 38.5 | 9.0 |
|  | average | 22.84 | 1.04 | 50.1 | 12.0 |

**Table S3** The photovoltaic performance of the devices with different perovskite film thickness based on Li_2_CO_3_ modified C-SnO_2_ as ETL

| **Device type** | ***V*_oc_ (V)** | ***J*_sc_ (mA/cm^2^)** | **FF (%)** | **PCE (%)** |
| --- | --- | --- | --- | --- |
| **1.0 M FA_0.3_MA_0.7_PbI_3_** | 1.138 | 23.19 | 68.2 | 18.0 |
| **1.2 M FA_0.3_MA_0.7_PbI_3_** | 1.152 | 23.41 | 69.7 | 18.8 |
| **1.4 M FA_0.3_MA_0.7_PbI_3_** | 1.142 | 23.67 | 70.6 | 19.1 |

**Table S4** The photovoltaic performance of device with the structure of Li_2_CO_3_@C-SnO_2_/perovskite/Spiro-OMeTAD/Au

| **Device type** | ***V*_oc_ (V)** | ***J*_sc_ (mA/cm^2^)** | **FF (%)** | **PCE (%)** |
| --- | --- | --- | --- | --- |
| **Li_2_CO_3_@SnO_2(spin)_** | 1.154 | 24.62 | 78.4 | 22.3 |

**Table S5** The photovoltaic performance of device with Li_2_CO_3_@SnO_2(spin)_ as ETL

| **Device type** | ***V*_oc_ (V)** | ***J*_sc_ (mA/cm^2^)** | **FF (%)** | **PCE (%)** |
| --- | --- | --- | --- | --- |
| **Li_2_CO_3_@SnO_2(spin)_** | 1.018 | 22.89 | 64.4 | 15.0 |

**Table S6** The photovoltaic performance of control and target devices measured under LED illumination at 1000 lux with the color temperature of 3000 K

| **Device type** | ***V*_oc_ (V)** | ***J*_sc_ (mA/cm^2^)** | **FF (%)** | ***P*_max_ (μW/cm^2^)** | ***P*_in_ (μW/cm^2^)** | **PCE (%)** |
| --- | --- | --- | --- | --- | --- | --- |
| **Control** | 0.885 | 0.104 | 68.4 | 63.0 | 281 | 22.4 |
| **Target** | 0.961 | 0.129 | 72.9 | 90.6 | 280 | 32.4 |

**Table S7** The photovoltaic performance of target devices measured under LED illumination at various intensities with the color temperature of 3000 K

| **Intensity (Lux)** | ***V*_oc_ (V)** | ***J*_sc_ (mA/cm^2^)** | **FF (%)** | ***P*_max_ (μW/cm^2^)** | ***P*_in_ (μW/cm^2^)** | **PCE (%)** |
| --- | --- | --- | --- | --- | --- | --- |
| **500** | 0.936 | 0.065 | 72.8 | 44.2 | 136 | 32.5 |
| **1000** | 0.961 | 0.129 | 72.9 | 90.6 | 280 | 32.4 |
| **2000** | 0.989 | 0.258 | 72.7 | 186 | 560 | 33.2 |

**Table S8** The photovoltaic performance of target devices measured under LED illumination at 1000 lux with the color temperature of 3000 K, 4000 K, and 5500 K

| **Color temperature (K)** | ***V*_oc_ (V)** | ***J*_sc_ (mA/cm^2^)** | **FF (%)** | ***P*_max_ (μW/cm^2^)** | ***P*_in_ (μW/cm^2^)** | **PCE (%)** |
| --- | --- | --- | --- | --- | --- | --- |
| **3000** | 0.961 | 0.129 | 72.9 | 90.6 | 280 | 32.4 |
| **4000** | 0.960 | 0.136 | 72.0 | 94.2 | 285 | 33.1 |
| **5500** | 0.957 | 0.135 | 71.1 | 91.8 | 301 | 30.5 |

**Table S9** The photovoltaic performance of target devices measured under LED illumination at 2000 lux with the color temperature of 3000 K and 5500 K

| **Color temperature (K)** | ***V*_oc_ (V)** | ***J*_sc_ (mA/cm^2^)** | **FF (%)** | ***P*_max_ (μW/cm^2^)** | ***P*_in_ (μW/cm^2^)** | **PCE (%)** |
| --- | --- | --- | --- | --- | --- | --- |
| **3000** | 0.989 | 0.258 | 72.7 | 186 | 560 | 33.2 |
| **5500** | 0.978 | 0.225 | 72.0 | 159 | 500 | 31.7 |

**Table S10** Low-light-intensity photovoltaic performance of perovskite solar cells reported in the literature and in this work.

| **Device structure** | **Color temperature (K)** | **Light intensity (Lux)** | ***V*_oc_ (V)** | ***J*_sc_ (mA/cm^2^)** | **FF (%)** | **PCE (%)** | **Active area (cm^2^)** | **Refs.** |
| --- | --- | --- | --- | --- | --- | --- | --- | --- |
| FTO/c-TiO_2_/mp-TiO_2_/MAPbI_3−x_Cl_x_/GC@MAPbI_3−x_Cl_x_(deposited using the layer-to-layer approach)/Carbon | / | 200 | 0.9322 | 0.03765 | 59.55 | 20.90 | 88 | [47] |
| FTO/(c-TiO_2_/m-TiO_2_/m-ZrO_2_/Carbon)/MAPbI_3_ | / | 1000 | 0.79 | 0.1323 | 78 | 23.4 | 0.64 | [48] |
| IZO/SnO_2_/Cs_0.17_FA_0.83_Pb(I_0.7_Br_0.3_)_3_/Carbon | / | 1000 | 0.988 | 0.1337 | 68.8 | 23.1 | / | [49] |
| FTO/SnO_2_/MAPbI_3_/Carbon | white CFL | 1000 | 0.82 | 0.13989 | 54.98 | 22.37 | 0.25 | [50] |
| **FTO/SnO_2_/Li_2_CO_3_/FA_0.3_MA_0.7_PbI_3_/Carbon** | **3000** | **2000** | **0.989** | **0.258** | **72.7** | **33.2** | **0.09** | **This work** |
